# Generation and Dietary Modulation of Anti-Inflammatory Electrophilic Omega-3 Fatty Acid Derivatives

**DOI:** 10.1371/journal.pone.0094836

**Published:** 2014-04-15

**Authors:** Chiara Cipollina, Sonia R. Salvatore, Matthew F. Muldoon, Bruce A. Freeman, Francisco J. Schopfer

**Affiliations:** 1 Fondazione Ri.Med, Palermo, Italy; 2 Department of Pharmacology and Chemical Biology, University of Pittsburgh School of Medicine, Pittsburgh, Pennsylvania, United States of America; 3 Department of Medicine, University of Pittsburgh School of Medicine, Pittsburgh Pennsylvania, United States of America; University of Milan, Italy

## Abstract

Dietary ω-3 polyunsaturated fatty acids (PUFAs) decrease cardiovascular risk via suppression of inflammation. The generation of electrophilic α,β-unsaturated ketone derivatives of the ω-3 PUFAs docosahexaenoic acid (DHA) and docosapentaenoic acid (DPA) in activated human macrophages is catalyzed by cyclooxygenase-2 (Cox-2). These derivatives are potent pleiotropic anti-inflammatory signaling mediators that act via mechanisms including the activation of Nrf2-dependent phase 2 gene expression and suppression of pro-inflammatory NF-κB-driven gene expression. Herein, the endogenous generation of ω-3 PUFAs electrophilic ketone derivatives and their hydroxy precursors was evaluated in human neutrophils. In addition, their dietary modulation was assessed through a randomized clinical trial.

**Methods:**

Endogenous generation of electrophilic omega-3 PUFAs and their hydroxy precursors was evaluated by mass spectrometry in neutrophils isolated from healthy subjects, both at baseline and upon stimulation with calcium ionophore. For the clinical trial, participants were healthy adults 30–55 years of age with a reported EPA+DHA consumption of ≤300 mg/day randomly assigned to parallel groups receiving daily oil capsule supplements for a period of 4 months containing either 1.4 g of EPA+DHA (active condition, n = 24) or identical appearing soybean oil (control condition, n = 21). Participants and laboratory technicians remained blinded to treatment assignments.

**Results:**

5-lypoxygenase-dependent endogenous generation of 7-oxo-DHA, 7-oxo-DPA and 5-oxo-EPA and their hydroxy precursors is reported in human neutrophils stimulated with calcium ionophore and phorbol 12-myristate 13-acetate (PMA). Dietary EPA+DHA supplementation significantly increased the formation of 7-oxo-DHA and 5-oxo-EPA, with no significant modulation of arachidonic acid (AA) metabolite levels.

**Conclusions:**

The endogenous detection of these electrophilic ω-3 fatty acid ketone derivatives supports the precept that the benefit of ω-3 PUFA-rich diets can be attributed to the generation of electrophilic oxygenated metabolites that transduce anti-inflammatory actions rather than the suppression of pro-inflammatory AA metabolites.

**Trial Registration:**

ClinicalTrials.gov NCT00663871

## Introduction

The dietary intake of ω-3 polyunsaturated fatty acids (PUFAs) provides a significant reduction in adverse cardiovascular events and death [1]–[4]. Thus, the American Hearth Association and the European Society of Cardiology recommend a daily dose of approximately 1 g for secondary prevention [5]. ω-3 PUFAs are also effective in reducing the inflammatory phenotype in other chronic degenerative diseases and acute events [2], [6]–[8].

Among the proposed mechanisms of action through which ω-3 PUFAs mediate clinical benefits, the formation of bioactive oxygenated metabolites may be the most relevant. The enzymes cyclooxygenase-2 (Cox-2) and lipoxygenases (LOs) in part sustain the onset of inflammation by converting the ω-6 PUFA arachidonic acid (AA) into pro-inflammatory eicosanoids. These oxygenases also convert ω-3 PUFAs into bioactive lipid mediators that promote the resolution of inflammation [8]–[11]. Recently, the link between dietary intake of ω-3 PUFAs and the formation of oxygenated derivatives has been explored showing a positive correlation [12]–[19].

Among oxygenated PUFAs, electrophilic α,β-unsaturated ketone derivatives are of interest because they a) are end-products of hydroxylated fatty acid metabolism, b) display reversible electrophilic reactivity with nucleophilic protein targets via Michael addition reaction and c) modulate the activity of critical inflammatory and host defense signaling pathways [20]. In this regard, electrophilic PUFAs activate the Nrf2-dependent anti-oxidant response, are partial agonists of the nuclear lipid receptor PPARγ, and suppress pro-inflammatory NF-κB signaling at multiple levels [9], [21]–[23]. Recently, a method has been developed for the mass spectrometric detection and quantification of electrophilic fatty acids, based on the use of the β-mercaptoethanol (BME) as nucleophilic bait [24]. Its use in profiling electrophilic fatty acid generation by activated macrophages led to the discovery of Cox-2-dependent generation of 13- and 17-oxo-derivatives of docosahexaenoic (DHA) and docosapentaenoic acid (DPA)[9]. These electrophilic ketone derivatives mediate pleitropic anti-inflammatory actions via modulation of transcriptional regulatory protein function thus representing a new class of endogenous lipid mediators.

Neutrophils are the most abundant white blood cells and, as early responders to bacterial infections, are critical in shaping the inflammatory milieu by releasing AA-derived 5-LO metabolites including leukotriene B_4_ (LTB_4_), 5-hydroxy-eicosatetraenoic acid (5-OH-ETE) and 5-oxo-ETE [25], [26]. The formation of electrophilic 5-oxo-EPA by 5-LO in neutrophils supplemented with EPA antagonizes the pro-inflammatory actions of AA-derived metabolites [27]. Of note, the endogenous generation of 5-oxo-EPA and other electrophilic α,β-unsaturated ketone derivatives of ω-3 PUFAs has not been reported, most likely because of their facile Michael addition to cellular and plasma nucleophiles, which makes it difficult to detect these species in “free form” by traditional organic and/or solid phase extraction methods.

Herein, the endogenous formation of electrophilic α,β-unsaturated ketone derivatives of DHA, EPA and DPA is reported in neutrophils isolated from healthy subjects. Specifically, 5-LO-dependent generation of 7-oxo-DHA, 7-oxo-DPA, 5-oxoEPA and their hydroxy precursors is reported. Moreover, the effects of EPA and DHA consumption at a clinically-relevant dose (1.4 g/day) on the production of these ω-3 PUFA ketone derivatives was evaluated in healthy human subjects participating in a randomized, placebo-controlled clinical trial. There was a significant increase in levels of electrophilic ω-3 PUFA ketone derivatives in neutrophils under both basal and activated conditions, with no significant change in AA-derived metabolites. It is proposed that these reactive products transduce the salutary actions of ω-3 PUFAs by modifying the activity of electrophile-sensitive transcription factors that regulate the expression of inflammatory and metabolic-related genes. This report follows CONSORT guidelines for clinical trials [28].

## Materials and Methods

### Ethics statement

All subjects recruited for the first part of the work provided written informed consent, as approved by the University of Pittsburgh Investigational Review Board (IRB #PRO07040037). For the randomized clinical trial (ClinicalTrials.gov #NCT00663871) ethics oversight was provided by the University of Pittsburgh Human Subjects IRB and an expert external monitor.

### Materials

The following lipid standards were purchased from Cayman Chemical: 5-oxo-6E,8Z,11Z,14Z-eicosatetraenoic-6,8,9,11,12,14,15-d7 acid (5-oxoETE-d7, ≥99% deuterated product), 17-oxo-4Z,7Z,10Z,13Z,15E,19Z-docosahexaenoic acid (17-oxo-DHA, ≥98%), 17-oxo-7Z,10Z,13Z,15E,19Z-docosapentaenoic acid (17-oxo-DPA, ≥98%), (±)13-hydroxy-4Z,7Z,10Z,14E,16Z,19Z-docosahexaenoic acid (13-OH-DHA, >98%), (±)7-hydroxy-4Z,8E,10Z,13Z,16Z,19Z-docosahexaenoic acid (7-OH-DHA, >98%), 5-oxo-6E,8Z,11Z,14Z-eicosatetraenoic acid (5-oxoETE, >95%), 5S-hydroxy-6E,8Z,11Z,14Z-eicosatetraenoic acid (5-OH-ETE, >98%), (±)-5-hydroxy-6E,8Z,11Z,14Z,17Z-eicosapentaenoic acid (5-OH-EPA, >98%). Docosahexaenoic acid (DHA, >99%) and docosapentaenoic acid (DPA, >99%) were purchased from NuCheck Prep, Inc.

### Isolation of neutrophils

The blood of healthy adult volunteers was collected in EDTA-coated tubes and layered over an equal volume of Polymorphprep (Axis-Shield). Neutrophils were isolated by one-step density gradient centrifugation following manufacturer's instructions (http://www.axis-shield-density-gradient-media.com). Residual erythrocytes were lysed by resuspending pellets at 5°C in H_2_O for 30 sec, followed by addition of one vol 0.3 M NaCl. Cells were centrifuged, resuspended in Hanks' Balanced Salt Solution (HBSS) supplemented with 1.2 mM CaCl_2_ and 0.811 mM MgSO_4_, counted using a hemocytometer and neutrophil concentrations adjusted to 20×10^6^ cells/ml.

### Neutrophil stimulation and sample collection

Neutrophils were stimulated with A23187 (5 µM) or phorbol myristate acetate (PMA, 2 µM) and incubated at 37°C for the indicated times. Time 0 was collected before the stimulus was added. Where indicated, DHA or DPA was added before the stimulus at a final concentration of 30 µM. For inhibition experiments, the 5-LO activating protein (FLAP) inhibitor MK-886 was added at a final concentration of 500 nM [29], cells were incubated 15 min at 37°C and stimulated as indicated. Samples (100 µl) were collected at the indicated time points and centrifuged at 4°C. The pellet was resuspended in 20 µl 50 mM phosphate buffer, pH 7.4, snap frozen in liquid nitrogen and stored at -80°C until further processing.

### Capture of electrophilic fatty acid derivatives by BME reaction and LC-MS/MS analysis

For detection of PUFA electrophilic derivatives, samples were treated with 500 mM BME in presence of the internal standard 5-oxo-ETE-d7 (final concentration 2.5 ng/ml) for 2 h at 37°C [9], [24]. Reactions were stopped by addition of 4 volumes cold acetonitrile/1% formic acid, protein precipitates were removed by centrifugation and supernatants collected for mass spectrometry (MS) analysis. Fatty acids were resolved by reversed-phase high performance liquid chromatography (RP-HPLC) using 150×2 mm C18 Luna column (3 µm, Phenomenex) with a flow rate of 250 µl/min using the following gradient conditions: hold at 35% B for 3 min, increase to 90% B during 43 min, increase to 100% B and hold for 6 min to then re-equilibrate at initial conditions for 8 min. Analysis and quantification of BME adducts of electrophilic fatty acids and free fatty acids OH-derivatives were performed in the negative ion mode using an API-5000 (Applied Biosystems/MDS Sciex) mass spectrometer equipped with an electrospray ionization (ESI) probe in multiple reaction monitoring (MRM) scan mode. The following settings were used: declustering potential -50 V and collision energy -17 V. Zero grade air was used as source gas, and N_2_ was used in the collision chamber. Data were acquired and analyzed using Analyst 1.4.2 software (Applied Biosystems, Framingham, MA).

### Electrophilic fatty acid quantification and accurate mass determinations

Standard curves were generated using 5-OH-EPA, 5-OH-ETE, 7-OH-DHA and the BME addition products of 17-oxo-DHA, 17-oxo-DPA and 5-oxo-ETE using 5-oxo-ETE-d7 as internal standard by plotting area ratio (analyte/IS) vs concentration ratio (analyte/IS). High resolution mass determination and structural characterization was performed on a HPLC-coupled Velos Orbitrap hybrid mass spectrometer (Thermo Fisher Scientific, Bremen, Germany) equipped with a heated electrospray ionization (HESI) probe.

### In vitro enzymatic oxidation of omega-3 fatty acids

Human recombinant 5-lipoxygenase (0.7 U, Cayman Chemicals) was added to 50 mM Tris-HCl buffer, pH 7.4, containing 2 mM CaCl_2_ and 2 mM ATP, in the presence of 30 µM DHA or DPA. Reaction mixtures were incubated at 37°C for 15 min and reactions stopped by adding two volume acetonitrile chilled to 5°C. Enzyme was removed by precipitation, and supernatants were analyzed by LC-MS/MS.

### Clinical trial design

The randomized clinical trial herein reported is a substudy of a larger parent cross-sectional study: Adult Health and Behavior, Phase 2 (AHAB-2). Participants to AHAB-2 were initially recruited using mass mailing in the greater Pittsburgh metropolitan area between June 2008 and August 2011. Participants were required to be 30–54 years of age and employed at least 25 hours per week. Exclusion criteria included atherosclerotic disease (e.g., myocardial infarction or revascularization), history of psychosis (schizophrenia or bipolar disorder), chronic hepatitis, chronic kidney disease, chronic lung disease requiring daily drug treatment, stage 2 hypertension, alcohol consumption greater than 35 alcoholic drinks per week and, in women, pregnancy or lactation. Persons prescribed any cardiovascular, psychotropic, glucocorticoid, lipid-lowering, insulin or weight-loss medications were also excluded, as were those taking fish oil, algae, algal oil, or DHA supplements.

**Figure 1:**
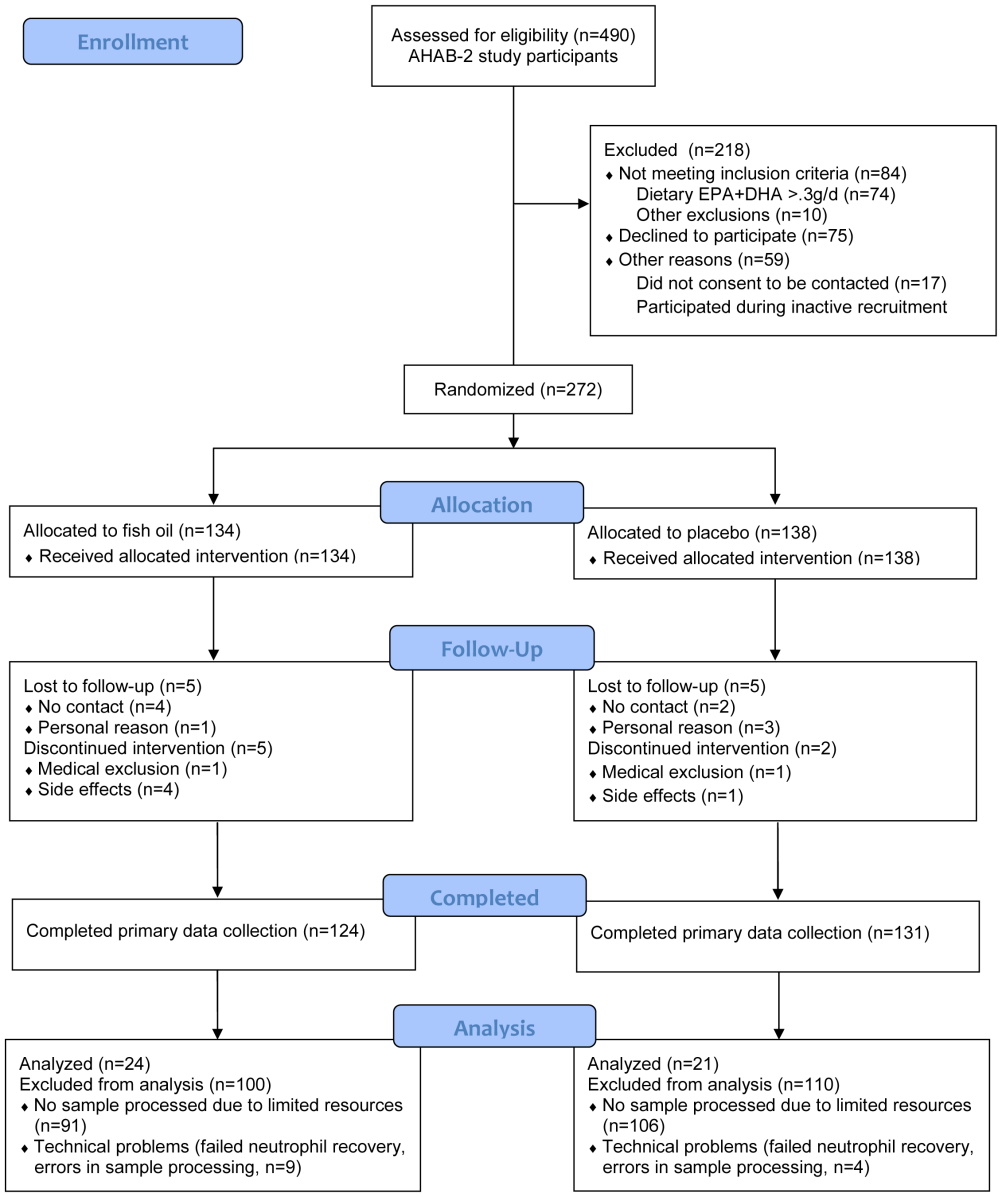
CONSORT flowchart of the study AHAB-2. Patients recruited for the present substudy are indicated at the bottom of the flow diagram under the heading “Analysis”.

Individuals who completed AHAB2 were invited to be screened for enrollment in a randomized and placebo-controlled trial of fish oil supplementation. (See Figure 1, CONSORT flow chart.) The additional exclusion criteria for the trial were allergy to fish and estimated daily dietary consumption of EPA and DHA combined greater than 300 mg/day. Eligible volunteers were assigned in an allocation ratio of 1∶1 to parallel groups receiving either daily fish oil supplements or matching soybean oil placebo capsules for 4 months. Treatment assignment was randomized, utilizing a computerized minimization algorithm considering gender, race (Black, Non-black) and age stratum (<45, >/ = 45) measured at baseline. Fish oil supplementation consisted of two 1000 mg capsules, with each capsule containing 500 mg EPA, 200 mg DHA, 10 IU of vitamin E and mint flavor. Placebo supplementation consisted of two 1000 mg capsules of soybean oil, each also containing 10 IU of vitamin E and mint flavor. Participants and laboratory technicians remained blinded to treatment assignment. The primary outcomes of the main clinical trial were circulating markers of systemic inflammation; autonomic control of heart rate; measures of cognitive functioning; and self-report, laboratory-based, and interview-based measures of affect, hostility, anger, impulsivity, and aggression (The protocol for this trial and supporting CONSORT checklist are available as supporting information; see Checklist S1 and Protocol S1). These data are being prepared for publication separately. The trial is registered at ClinicalTrials.gov (Evaluating the effects of omega-3 fatty acids on heart disease and behavior; NCT00663871).

The current report describes the trial's secondary outcomes related to the clinical generation and dietary modulation of electrophilic ω-3 fatty acid derivatives. To this purpose, samples were collected from a small subset of study participants recruited between January 2010 and December 2011 (see Figure 1). Baseline characteristics of the subpopulation used for the present study did not differ from those of the whole study population (Table S1). The sample size for this substudy was determined based on the availability of resources to conduct the specialized laboratory measures.

### Analysis of red blood cells (RBC) fatty acid composition

For RBC fatty acid analysis, hemoglobin-free RBC ghost membranes were prepared as previously described and stored at -70°C until further use [30]. Lipids were extracted from RBC ghost membranes [31] and fatty acid methyl esters (FAME) were prepared using methanolic KOH reagent [32]. Diheptadecanoyl lecithin (Matraya, Inc.) was used as an internal standard. Quantitative determination of fatty acid distribution was performed by capillary gas chromatography with flame ionization detector (GC-FID) using a Hewlett-Packard capillary gas chromatograph (Model 5890 Series II) equipped with a hydrogen flame ionization detector. The method used for the analysis has been previously described [33].

### Data analysis

The results are expressed as mean ± standard error (SE). Statistical analysis of pair wise comparison was done by applying the non-parametric Mann–Whitney–Wilcoxon test using the software StatView. A p-value<0.05 was considered statistically significant.

## Results

### Generation of electrophilic α,β-unsaturated ketone derivatives from DHA and DPA in human neutrophils

**Figure 2:**
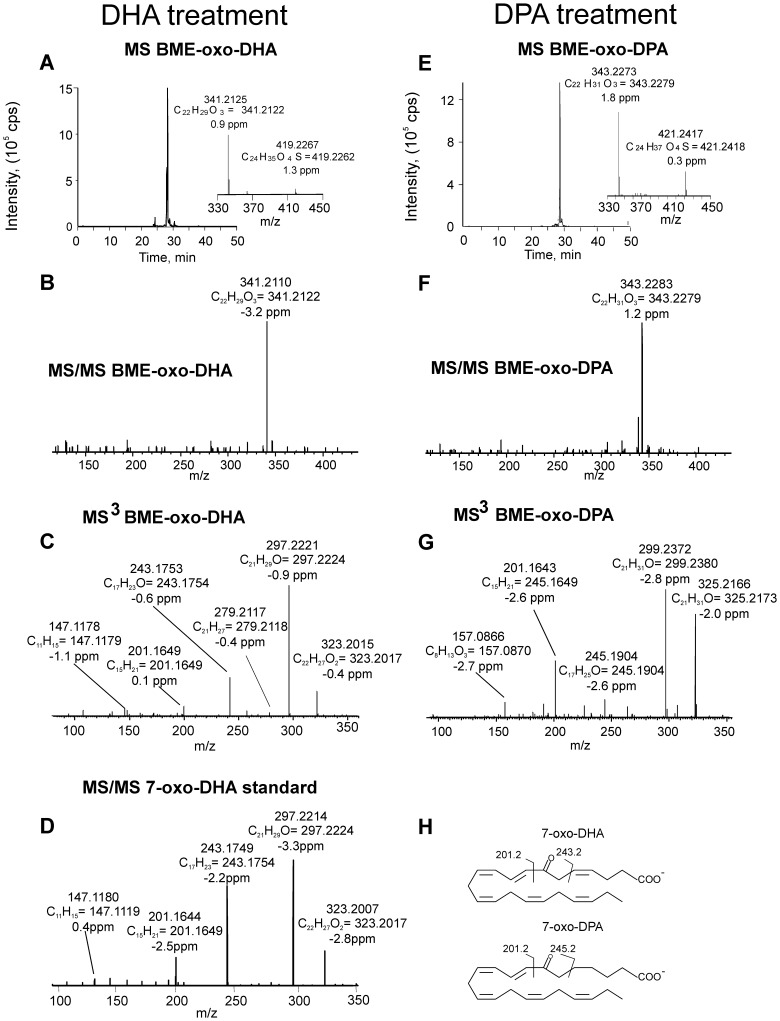
Generation of 7-oxo-DHA and 7-oxo-DPA by neutrophils supplemented with DHA or DPA and stimulated with calcium ionophore. Freshly isolated neutrophils were supplemented with 30 µM DHA (A–D) or DPA (E–G) and stimulated with calcium ionophore. Samples were collected at 15 min and cell extracts were reacted with 500 mM BME for detection of electrophilic fatty acids. (A,E) Chromatograms were acquired following the neutral loss of BME using the following transitions: 419.2 to 341.2(A) and 421.2 to 343.2 (E). Insets show mass spectra with respective BME β-elimination due to in source fragmentation. (B,F) Product ion analysis of BME-oxo-DHA (m/z 419.2, B) and BME-oxo-DPA (m/z 421.2, F); (C,G) MS/MS analysis of the free oxo-DHA (C) and oxo-DPA (G) generated by in-source fragmentation upon respective BME neutral loss; (D) MS/MS analysis of 7-oxo-DHA synthetic standard; (H) chemical structure and fragmentation pattern of 7-oxo-DHA and 7-oxo-DPA. 7-oxo-DHA standard was enzymatically synthesized by incubating 20 µM 7-OH-DHA with 3α-hydroxysteroid dehydrogenase in the presence of 100 µM NAD+ for 10 min at 37°C.

**Figure 3:**
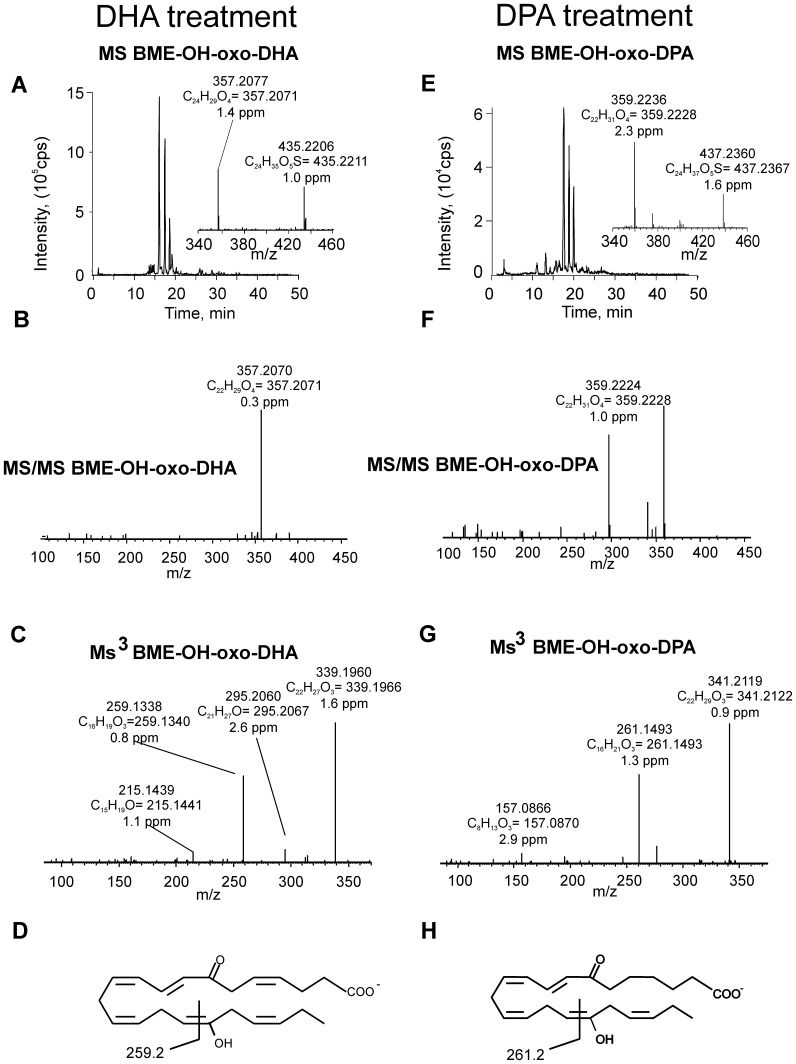
Generation of OH-oxo-DHA and OH-oxo-DPA by neutrophils supplemented with DHA or DPA and stimulated with calcium ionophore. Freshly isolated neutrophils were supplemented with 30 µM DHA (A-D) or DPA (E-H) and stimulated with calcium ionophore. Samples were collected at 15 min and cell extracts were reacted with excess BME for detection of electrophilic fatty acid derivatives. (A, E) Chromatograms showing elution of ions with m/z 435.2 (A) and 437.2 (E) and their respective BME β-elimination due to in source fragmentation (insets). (B, F) Product ion analysis of BME-OH-oxo-DHA (m/z 435.2, B) and BME-OH-oxo-DPA (m/z 437.2, F); (C, G) MS/MS analysis of the free OH-oxo-DHA (C) and OH-oxo-DPA (G) generated by in-source fragmentation upon respective BME neutral loss; (D, H) proposed chemical structure and fragmentation pattern of 17-OH-7-oxo-DHA (D) and 17-OH-7-oxo-DPA (H).

Freshly isolated human neutrophils were supplemented with 30 µM DHA or DPA and stimulated with calcium ionophore for 15 min. Cell extracts were treated with BME to capture electrophilic species and analyzed by HPLC-MS/MS. When neutrophils were supplemented with DHA, species displaying a neutral loss of 78 amu revealed a major parent ion with m/z 419.2 corresponding to BME addition to oxo-DHA (m/z 341.2+78) (Figure 2A). MS/MS analysis and accurate mass determination at the 2 ppm level confirmed the atomic composition of the parent and product ions (Figure 2A, B). Neutrophils supplemented with DPA presented a major ion with m/z 421.2, corresponding to BME addition to oxo-DPA that fragmented through the neutral loss of BME (collision-induced gas phase β-elimination reaction)(Figure 2E, F). For structural characterization, two different methodological approaches were used: a) MS/MS fragmentation of the ions generated from BME loss during in-source fragmentation (in source β-elimination of BME) and b) MS3 following the sequential loss of BME and fragmentation of the resulting product ion. Both methods gave similar results, although the overall efficiency and intensities obtained upon in-source fragmentation were usually higher (Figure 2C, G). Fragmentation of oxo-DHA (m/z 341.2) gave, in addition to the non-specific ions corresponding to loss of water (m/z 323.2) and loss of CO_2_ (m/z 297.2), two diagnostic fragment ions at m/z 201.2 and 243.2 (Figure 2C). These corresponded to the fragmentation pattern of 7-oxo-DHA as further confirmed by comparison with a synthetic standard (Figure 2D). Neutrophil-derived oxo-DPA (m/z 343.2) presented a similar fragmentation pattern that, in addition to the loss of water and CO_2_ (m/z 325.2 and 299.2, respectively), gave two diagnostic fragment ions at m/z 201.2 and 245.2 (Figure 2G, H), characteristic of 7-oxo-DPA. Formation of di-oxygenated electrophilic derivatives of DHA and DPA was also observed in neutrophils supplemented with the respective omega-3 fatty acid precursors. In particular, the formation of oxo-OH-DHA was observed in neutrophils supplemented with DHA. This species reacted with BME, giving an adduct with m/z of 435.2 and, after fragmentation-induced neutral loss of BME, an ion with m/z 357.2 (Figure 3A, B). A similar chromatographic profile was observed for DPA-derived oxo-OH-derivatives in DPA-supplemented samples which gave an adduct with m/z 437.2 characterized by the major neutral loss of BME upon fragmentation and the detection of the free oxo-OH fatty acid with m/z 359.2 (Figure 3E, F). Accurate mass determination and MS/MS analysis of the in-source generated ion corresponding to the free fatty acid revealed the formation of a diagnostic fragment ion with m/z 259.1 from oxo-OH-DHA (elemental composition C_16_H_19_O_3_) and 261.1 from oxo-OH-DPA (elemental composition C_16_H_21_O_3_) (Figure 3C, G). These fragments contained the oxo- and the carboxy-group, inferring a specific fragmentation induced by the presence of an OH group at C-17 (Figure 3D, H).

**Figure 4:**
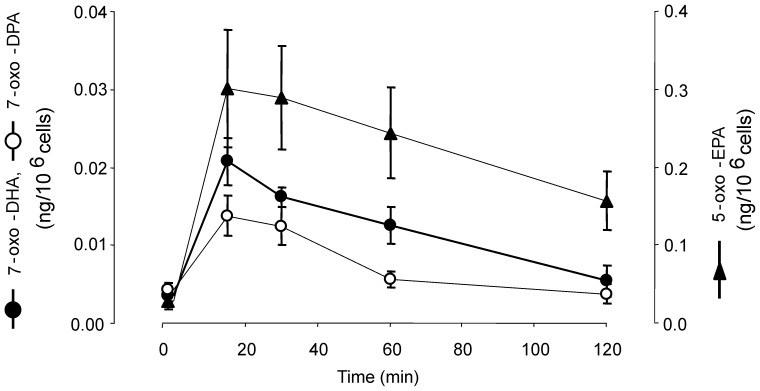
Time course of the generation of electrophilic 7-oxo-DHA, 7-oxo-DPA and 5-oxo-EPA by activated neutrophils. Freshly isolated neutrophils from three independent healthy subjects were stimulated with calcium ionophore, samples were collected at the indicated time points and cell extracts were reacted with 500α,β-unsaturated omega-3-derived fatty acids by LC-MS/MS.

**Figure 5:**
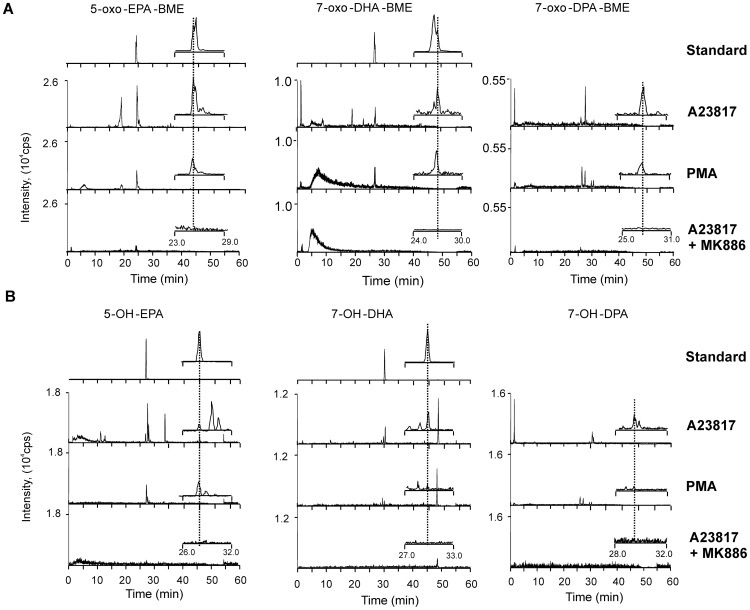
Endogenous generation of 5-oxo-EPA, 7-oxo-DHA, 7-oxo-DPA and their hydroxy precursors by activated neutrophils. Freshly isolated neutrophils were stimulated for 15-MS/MS in MRM mode. (A) BME adducts of 5-oxo-EPA, 7-oxo-DHA and 7-oxo-DPA were measured following loss of BME. (B) For the measurement of non-electrophilic hydroxy derivatives the following transitions were used: 317.2/299.2 (5-OH-EPA), 343.2/281.2 (7-OH-DHA), 345.2/327.2 (7-OH-DPA). Where available, chromatograms of synthetic standards are also reported. 7-oxo-DHA and 5-oxo-EPA standards were enzymatically synthesized by incubating 20 µM of OH-precursors with 3α-hydroxysteroid dehydrogenase, in presence of 100 µM NAD+ for 10 min at 37°. Insets show a magnified view of chromatograms. Std, standard.

For sensitive quantification of electrophilic α,β-unsaturated ketone derivatives, MS analysis was performed following the loss of BME in multiple reaction monitoring (MRM) mode [24]. The generation of 7-oxo-DHA, 7-oxo-DPA and 5-oxo-EPA was measured over time in neutrophils stimulated with calcium ionophore. Extents of ketone metabolite formation were greatest at 15 min and afterwards returned towards basal levels (Figure 4). To further investigate the endogenous formation of ω-3 fatty acid-derived electrophilic species after protein kinase C (PKC) activation, phorbol myristate acetate (PMA) was added in the absence of external fatty acid supplementation. As reported in Figure 5, neutrophils stimulated with PMA generated 5-oxo-EPA, 7-oxo-DHA and 7-oxo-DPA and their hydroxy precursors. All of these species co-eluted with those generated by calcium ionophore-stimulated neutrophils and with available synthetic standards.

### 5-lipoxygenase is required for generation of 7-oxo-DHA and 7-oxo-DPA by neutrophils

**Figure 6:**
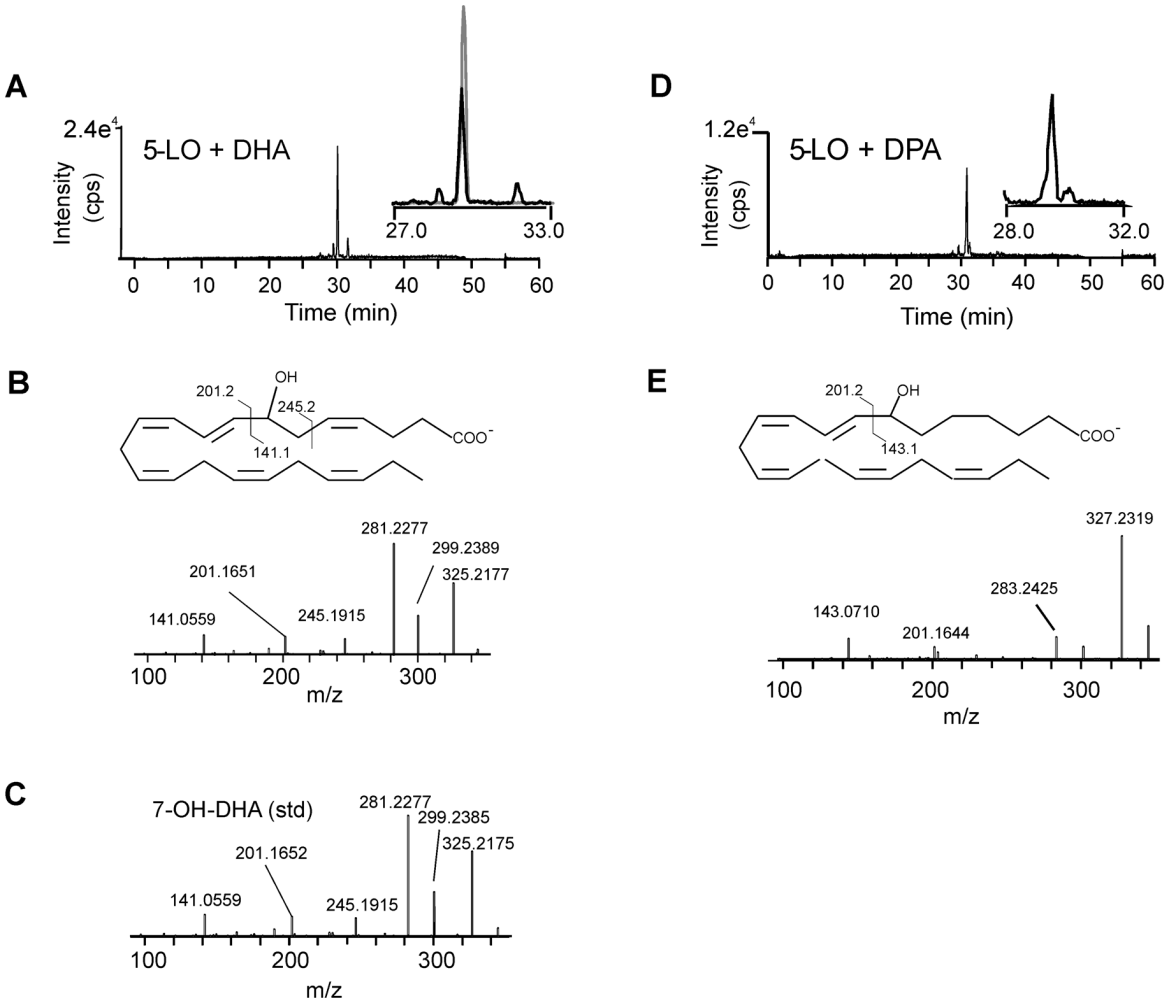
In vitro synthesis of 7-OH-DHA and 7-OH-DPA from DHA and DPA precursors by human recombinant 5-LO. Human recombinant 5-LO was incubated with 30 µM DHA (A-C) or DPA (D-E) in the presence of 2 mM ATP at 37°C for 15 min and ion product formation was analyzed by LC-MS/MS using high resolution mass spectrometry (Velos/Orbitrap (A, D)). For 7-OH-DHA the elution profile of the synthetic standard is reported as grey line in the inset (A). (B, E) Product ion spectra, chemical structures and interpretation of fragmentation upon collision induced dissociation. (C) Mass spectrum of 7-OH-DHA chemical standard.

To assess whether 5-LO was required for the generation of the electrophilic 7-oxo-DHA and 7-oxo-DPA, neutrophils were pretreated with the 5-LO specific inhibitor MK-886 and formation of DHA- and DPA-oxo-derivatives and their hydroxy precursors was evaluated after stimulation with calcium ionophore. Inhibition of 5-LO completely suppressed the formation of 7-oxo-DHA and 7-oxo-DPA and their hydroxy precursors to an extent similar to what was observed for inhibition of 5-oxo-EPA and 5-OH-EPA generation (Figure 5). Product analysis of the *in vitro* reaction of human recombinant 5-LO with DHA and DPA showed that 5-LO primarily generates the 7-OH-derivative from both substrates, supporting that this enzyme mediates the first biosynthetic step in the formation of electrophilic 7-oxo-derivatives from 22 carbon PUFAs (Figure 6).

### Dietary intake of EPA and DHA increases the formation of ω-3 PUFA α,β-unsaturated ketone derivatives in neutrophils under basal conditions and upon activation

It was next investigated whether the administration of clinically-relevant EPA and DHA doses that are recommended for cardiovascular prevention [5] modulates the formation of electrophilic α,β-unsaturated ketone derivatives by neutrophils. To this purpose 45 healthy volunteers with <300 mg/day consumption of EPA and DHA received either fish oil (1 g/day EPA and 0.4 g/day DHA) or control administration of 2 g/day soybean oil. Participants in the two treatment groups did not differ in biometric characteristics, baseline dietary EPA+DHA consumption and baseline red blood cell (RBC) levels of EPA+DHA (Table 1). Regarding the use of aspirin or other nonsteroidal anti-inflammatory drugs (NSAIDs) at baseline, 2 persons reported taking aspirin (one in the control group and one in the fish oil group) and 16 persons reported other NSAIDs (7 in the control group and 9 in the fish oil group).

**Table 1:** Characteristics of clinical trial study population.

	Control	Treatment	p-value^a^
N	21	24	
Gender (% female)	67%	67%	0.99
Age (years)	43.3±6.9	44.8±7.8	0.51
BMI (Kg/m^2^)	27.4±4.9	28.4±6.3	0.58
Dietary EPA+DHA (mg/day)	97±66	102±70	0.83
Baseline EPA+ DHA in RBCs (mole %)	2.57±1.41	2.85±1.35	0.47
Post-supplementation EPA+ DHA in RBCs (mole %)	2.24±1.09	4.05±1.79	<.001

Continuous variables are reported as mean ± standard deviation.

BMI, body mass index; EPA, eicosapentaenoic acid; DHA, docosahexaenoic acid; RBCs, red blood cells.

a based on Chi-square statistic for gender and t-test for other measures.

**Figure 7:**
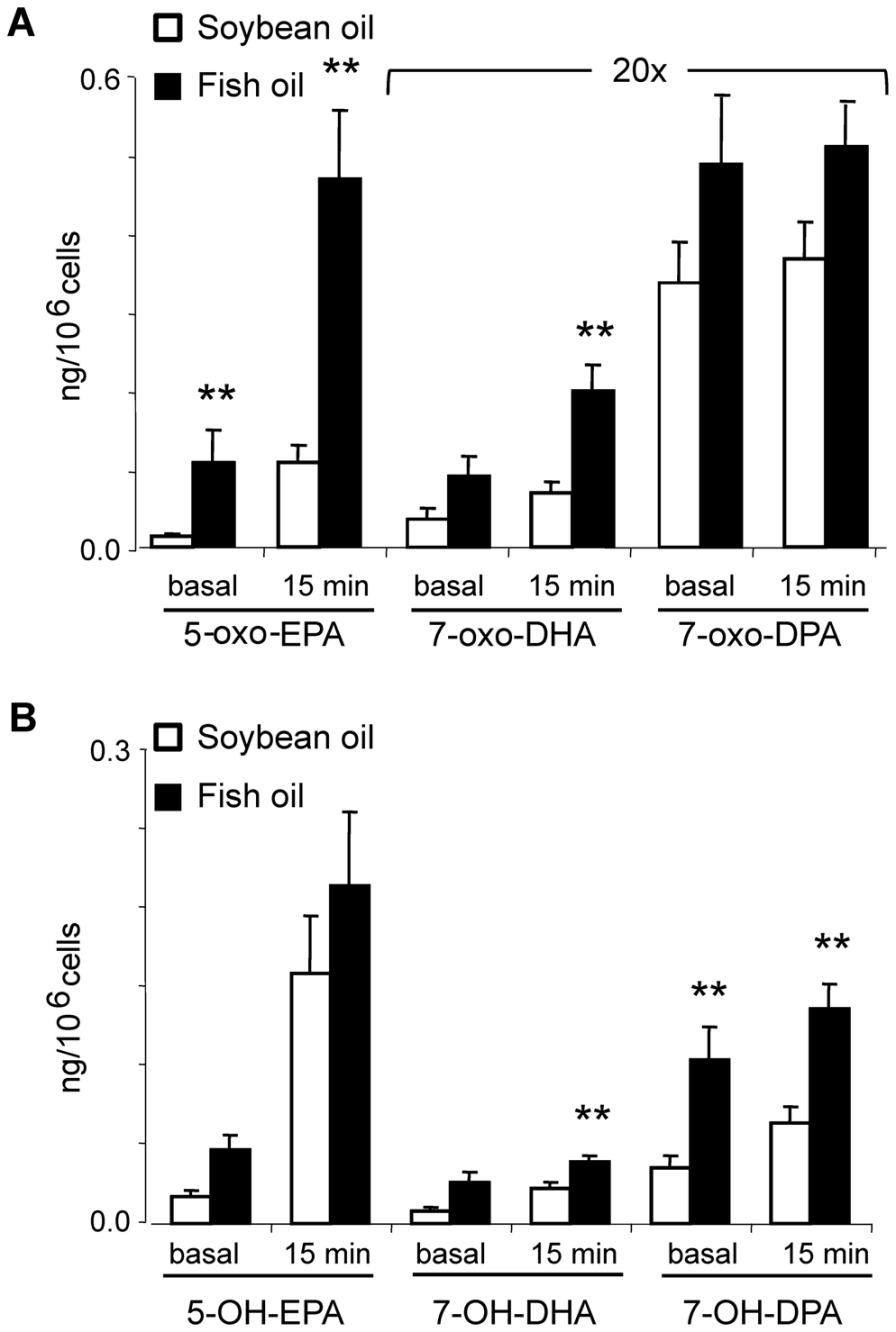
Dietary modulation of 5-oxo-EPA, 7-oxo-DHA and 7-oxo-DPA generation and their hydroxy precursors by neutrophils. Formation of 5-oxo-EPA, 7-oxo-DHA, 7-oxo-DPA (A) and their hydroxy precursors (B) was measured in freshly isolated neutrophils under basal conditions and after stimulation with calcium ionophore in healthy subjects consuming either control soybean oil (white bars) or a fish oil-supplemented diet (black bars). Neutrophils were stimulated for 15 min with calcium ionophore, cell extracts were reacted with 500 mM BME and the BME-adducted electrophilic oxo-fatty acids as well as non-electrophilic hydroxy fatty acids were quantified by LC-MS/MS. Data are expressed as mean ± SE, n = 21 and 24 for control and fish oil, respectively. The Mann–Whitney U test was used for pair wise comparisons of patients groups. **, p-value<0.005.

At the completion of the four month treatment period, fasting blood samples were obtained. Neutrophils were isolated from the freshly-drawn blood and endogenous levels of 5-oxo-EPA, 7-oxo-DHA, 7-oxo-DPA, 5-oxo-ETE and their hydroxy precursors were measured at basal conditions and 15 min after stimulation with calcium ionophore. The supplementation of EPA and DHA significantly increased the levels of oxygenated EPA and DHA derivatives in neutrophils, in particular 5-oxo-EPA and 7-oxo-DHA, both under basal conditions and after stimulation (Figure 7A). Mean 7-oxo-DPA levels slightly increased in the fish oil group, but this was not statistically significant. Dietary EPA+DHA supplementation increased 5-OH-EPA and 7-OH-DHA, precursors to electrophilic metabolites, to a lesser extent than electrophilic ketone derivatives (Figure 7B). Only 7-OH-DPA (and 7-OH-DHA after stimulation) accumulated to significantly greater levels in the fish oil group before and after calcium ionophore stimulation, inferring that further oxidation to the bioactive α,β-unsaturated ketone product was less efficient compared to 5-OH-EPA and 7-OH-DHA. The analysis of AA-derived 5-oxo-ETE, 5-OH-ETE and LTB4 metabolites under basal conditions and after neutrophil activation revealed no significant differences between the control and the fish oil group (Figure S1).

## Discussion

**Figure 8:**
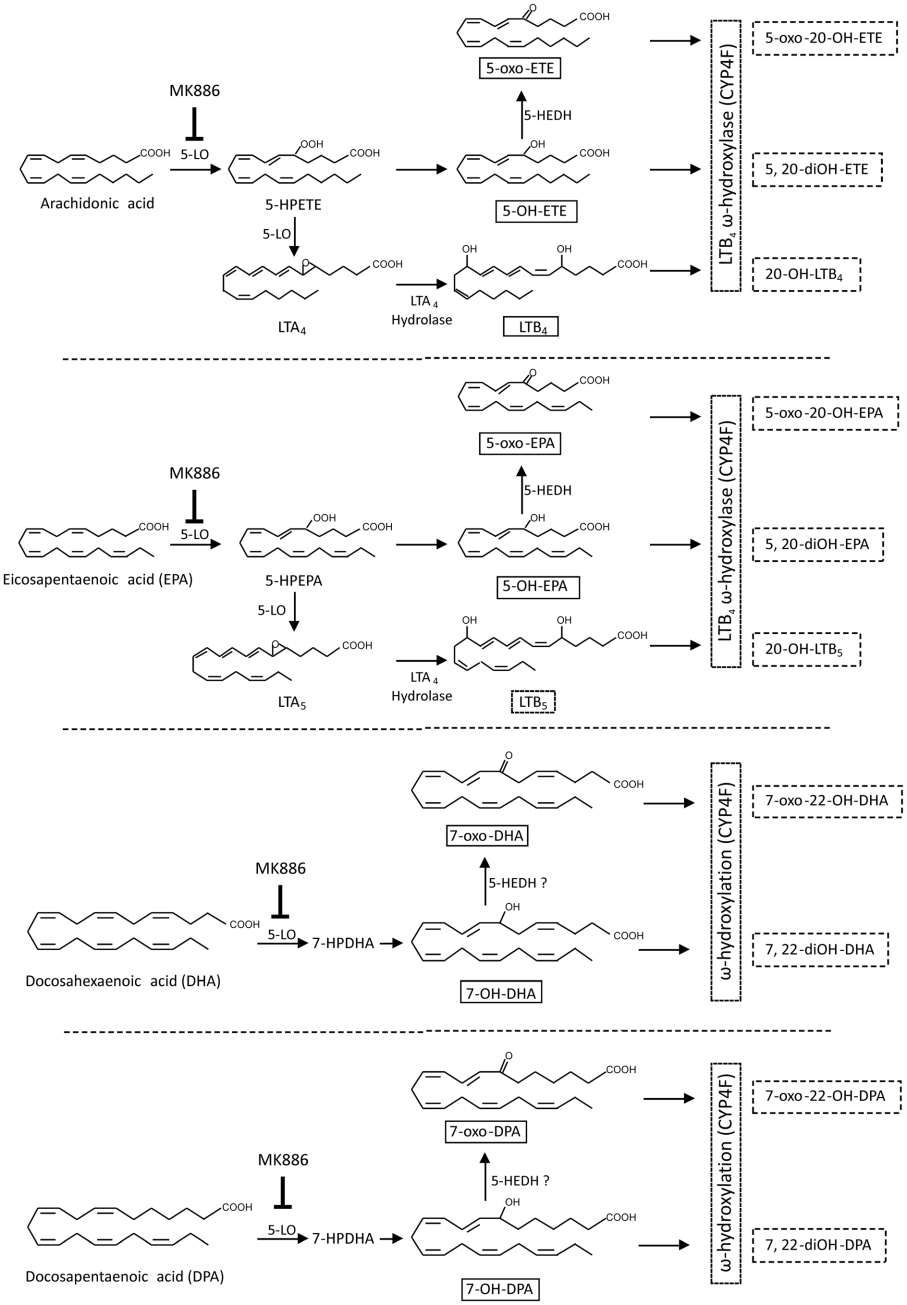
Enzymatic pathways leading to the formation of long chain PUFAs oxidized derivatives in activated neutrophils. Compounds indicated in solid boxes are those measured in the present study. Compounds indicated in dotted boxes are those that may be formed in activated neutrophils but were not measured in the present study. MK-886 is the 5-LO activating protein (FLAP) inhibitor used in this study. (5-LO, 5-lipoxygenase; 5-HEDH, 5-hydroxyeicosanoid dehydrogenase).

The generation of electrophilic α,β-unsaturated ketone derivatives of the ω-3 fatty acids EPA, DHA and DPA was mediated by neutrophils isolated from healthy subjects. The enzyme 5-LO catalyzed the synthesis of the hydroxy precursors 5-OH-EPA, 7-OH-DHA and 7-OH-DPA which were then oxidized to electrophilic α,β-unsaturated ketone derivatives by cellular dehydrogenases (Figure 8). The detection and quantification of these low molecular weight thiol-reactive signaling mediators by mass spectrometry was facilitated by the use of the nucleophilic BME as an electrophile-trapping bait. The supplementation of human volunteers with EPA and DHA for 4 months enhanced the generation of these bioactive neutrophil products under both basal conditions and after neutrophil activation. Notably, these conditions did not significantly impact the formation of pro-inflammatory AA-derived eicosanoids.

A transformative insight from this work is the observation that a diet rich in ω-3 EPA and DHA increases the formation of anti-inflammatory electrophilic α,β-unsaturated ketone fatty acid derivatives by neutrophils. Current understanding supports that electrophilic fatty acid oxygenation products mediate potent anti-inflammatory and cytoprotective signaling actions via both receptor-dependent and –independent mechanisms [9], [20]. These species signal by undergoing covalent Michael addition with nucleophilic targets such as cysteine and histidine residues of proteins. By reversibly adducting susceptible targets, a property defined by structural location and chemical reactivity, electrophilic PUFA derivatives modulate signaling pathways involved in the control of metabolic and inflammatory status of the cell. For example, suppression of pro-inflammatory NF-κB-regulated gene expression, partial agonist activity for the peroxisome proliferators-activated receptor gamma (PPARγ) and activation of Nrf2-regulated phase II gene expression account for many of the actions of these electrophilic ω-3 PUFA metabolites [9], [20], [21], [23], [34].

In vitro studies show that 5-oxo-EPA is generated by neutrophils after supplementation with EPA via consecutive enzymatic reactions catalyzed by 5-LO and 5-hydroxyeicosanoid dehydrogenase (5-HEDH) [27]. Herein, basal and dietary-enhanced levels of 7-oxo-DHA and 7-oxo-DPA and their respective hydroxy precursors are reported for the first time. Basal levels of these species were detected in neutrophils isolated from healthy subjects, and these levels increased after neutrophil activation by either calcium ionophore or PMA in the absence of *in vitro* or clinical ω-3 PUFA supplementation. The formation of these species in neutrophils was abolished upon treatment with the 5-lipoxygenase activating protein (FLAP) inhibitor MK-866, revealing a role for 5-LO in 7-OH-DHA and 7-OH-DPA synthesis. 5-HEDH, which displays broad substrate selectivity, is a likely candidate for the catalysis of 7-OH-DHA and 7-OH-DPA oxidation to α,β-unsaturated ketone derivatives [35]. Notably, 5-oxo-EPA reached much higher levels compared to oxo-DHA and oxo-DPA, despite the fact that levels of the hydroxy precursors were of the same order of magnitude (Figure 7). This suggests that 5-OH-EPA is converted into the ketone derivative more efficiently than 7-OH-DHA and 7-OH-DPA. These electrophilic products reached a maximum concentration 15 min after neutrophil stimulation and returned to baseline levels at later times (Figure 4). This kinetic profile for neutrophil generation of electrophilic fatty acids contrasts with activated macrophages, where Cox-2-dependent formation of 13- and 17-oxo-derivatives of DHA and DPA reached maximum levels 6 to 8 hr after stimulation [9]. The differing kinetics and electrophilic product distribution for neutrophils compared to macrophages reflect cellular differences in fatty acid oxygenation specificity and mechanisms of expression and regulation of 5-LO and Cox-2.

In addition to mono-oxygenated EPA and DHA products, chromatographic peaks corresponding to di-OH derivatives were detected in neutrophils that could correspond to LTB_5_ and other omega-hydroxylation derivatives (Figure 8). These species are of interest, but the low levels detected precluded any further characterization and quantification. Tri-OH species (termed lipoxins and resolvins) were undetectable. Importantly, high levels of electrophilic ions displaying m/z values corresponding to oxo-OH-EPA and oxo-OH-DHA were detected in fatty acid-supplemented neutrophils (Figure 3). Although these higher levels of electrophilic species may be attributed to the increased sensitivity of detection of oxygenated fatty acids by the BME-based analytical approach, the abundance of combined keto/hydroxy metabolites highlights the potential significance of these electrophilic species as electrophilic signaling mediators that can modulate adaptive gene expression during inflammation. The MS/MS fragmentation pattern of these species and high resolution mass determinations at the 2 ppm level indicated that the second OH-group was at carbon 17, inferring a 15-LO-catalyzed oxygenation product [36]. Since neutrophils do not express 15-LO, the 17-hydroxy products detected herein may arise from low levels of contaminating eosinophils present after Polymorphprep isolations [37].

The dietary supplementation of omega-3 PUFAs provides significant health benefits, by reducing the risk of cardiovascular mortality and sudden cardiac death [1]. There is also an associated reduction of plasma triglyceride levels, reduced risk of atrial fibrillation [38] and a variety of local and systemic anti-inflammatory actions of ω-3 PUFAs both in vitro and in vivo [39]–[42]. The suppression of AA-derived pro-inflammatory eicosanoids and an increased generation of bioactive oxygenated metabolites are the two most relevant mechanisms through which ω-3 PUFAs have been proposed to exert anti-inflammatory actions. While there is a positive correlation between dietary consumption of EPA and DHA and formation of oxygenated metabolites [13], [15], [16], [18], [19], [43], the effects of EPA and DHA consumption on AA metabolites levels are controversial and likely dependent on ω-3 PUFA dosing regimens. Several studies have evaluated the impact of dietary ω-3 PUFAs on the production of AA-derived eicosanoids and modulation of membrane lipid composition by lowering ω-6 / ω-3 ratios [12], [13], [15], [16], [18], [19], [44]–[47]. A decrease of AA-metabolite generation in human plasma as well as in activated neutrophils and macrophages is typically observed upon administration of high concentrations (∼5 g/day) of ω-3 PUFAs [13], [43], [46]. In the current study, no significant differences in the basal content or activated neutrophil formation of AA-derived eicosanoids was observed in both control and fish oil-supplemented groups (Figure S1).

The current data support the precept that the biochemical basis for the beneficial actions of dietary ω-3 PUFAs is the increased synthesis of bioactive oxygenated species rather than the suppression of pro-inflammatory AA-derived products. Furthermore, increased baseline levels of anti-inflammatory and electrophilic ω-3 PUFA derivatives, as reported herein for the fish oil-supplemented group, may limit inflammation and tissue damage. In this regard, both receptor-dependent and –independent signaling actions of fatty acid electrophiles would result in transcriptional suppression of genes involved in inflammatory propagation reactions and the activation of cytoprotective mechanisms [9], [40], [41], [48].

With regards to the clinical trial herein presented, some study limitations should be acknowledged. First, measurements were not obtained at baseline, before the beginning of dietary supplementation. Therefore, although unlikely due to randomization, the possibility that the observed differences between the two groups existed irrespective of fish oil supplementation cannot be excluded. Second, the study had a small sample size. Nevertheless, statistically significant differences were observed between the two study groups, in particular when analyzing oxidized derivatives of EPA and DHA. Finally, as these measures were obtained in the specified ex vivo preparations, it remains to be determined whether increased intake of EPA and DHA would affect electrophilic ω-3 fatty acid ketone derivative generation in vivo. Despites these limitations, findings herein reported are of great significance as they provide the first evidence from a controlled clinical trial that customary doses of dietary EPA and DHA increases the generation of electrophilic oxygenated derivatives with known anti-inflammatory properties.

In summary, we report the formation and dietary modulation of oxygenated electrophilic ω-3 PUFA ketone derivatives in neutrophils from healthy subjects. These findings support a role for dietary ω-3 PUFAs in exerting salutary cell signaling effects through the formation of electrophilic oxygenated derivatives. Furthermore, the results highlight the importance of neutrophils as first responders to sites of injury and inflammation and the function of electrophilic fatty acids in limiting the progression of inflammation and induction of tissue-protective responses.

## Supporting Information

Figure S1 **Endogenous generation of AA metabolites by neutrophils from subjects on a fish oil diet compared to the control group.** 5-oxo-ETE, 5-OH-ETE and LTB4 were measured in freshly isolated neutrophils under basal conditions and upon stimulation with calcium ionophore in healthy subjects consuming either control soybean oil (white bars) or a fish oil-supplemented diet (black bars). Neutrophils were stimulated for 15 min with calcium ionophore, cell extracts were reacted with BME and electrophilic fatty acids were quantified by LC-MS/MS. Data are expressed as mean ± SE, n = 21 and 24 for control and fish oil, respectively. The Mann–Whitney U test was used for pair-wise comparisons of patient groups. No significant differences were observed.
(TIF)

Table S1 **Baseline characteristics of study participants in comparison with remainder of total randomized trial population.**
(DOCX)

Protocol S1 **AHAB-II study and trial of fish oil supplementation.**
(DOC)

Checklist S1 **CONSORT 2010 checklist of randomized trial information.**
(DOC)
