# Supplementary material for: Generation and Dietary Modulation of Anti-Inflammatory Electrophilic Omega-3 Fatty Acid Derivatives
Source: PLoS One. 2014 Apr 15;9(4):e94836. doi: 10.1371/journal.pone.0094836 (PMC3988126; doi:10.1371/journal.pone.0094836)
Supplement: Table S1 — Baseline characteristics of study participants in comparison with remainder of total randomized trial population. (DOCX) [file pone.0094836.s002.docx]

| **Table S1.** Baseline characteristics of study participants in comparison with remainder of total randomized trial population | | | |
| --- | --- | --- | --- |
|  | **Study participants** | **Remainder of Trial population** | **p-value^a^** |
| N | 45 | 227 |  |
| Gender (% female) | 67% | 57% | >0.10 |
| Age (years) | 44.1 ± 7.36 | 42.5 ± 7.25 | >0.10 |
| BMI (Kg/m^2^) | 27.4 ± 4.86 | 26.9 ± 5.22 | >0.10 |
| Dietary EPA+DHA (mg/day) | 100 ± 67 | 109 ± 72 | >0.10 |
| EPA+DHA in RBCs (mole %) | 2.73 ± 1.37 | 3.00 ± 0.95 | >0.10 |
| Continuous variables are reported as mean ± SD. | | | |
| BMI, body mass index; EPA, eicosapentaenoic acid; DHA, docosahexaenoic acid; RBCs, red blood cells | | | |
| ^a^ based on Chi-square statistic for gender and t-test for other measures | | | |
