# Supplementary material for: Generation and Dietary Modulation of Anti-Inflammatory Electrophilic Omega-3 Fatty Acid Derivatives
Source: PLoS One. 2014 Apr 15;9(4):e94836. doi: 10.1371/journal.pone.0094836 (PMC3988126; doi:10.1371/journal.pone.0094836)
Supplement: Protocol S1 — AHAB-II study and trial of fish oil supplementation. (DOC) [file pone.0094836.s003.doc]

Overview

# **AHAB-II, which consists of three related substudies, will examine psychobiological dimensions of individual differences and variation in socio-environmental and nutritional exposures as correlates of heart disease risk factors and subclinical atherosclerosis.**

# **Substudy 1 seeks to establish whether psychosocial and lifestyle-related risk factors for cardiovascular disease are associated with individual differences in the reactivity of key neural structures underlying emotional processing and appetitive motivation.**

# **Substudy 2 will investigate stress-related features of the social environment (in both occupational and home contexts), tracking individuals' behavioral and biological reactions in real time and elucidating pathways to preclinical disease.**

# **Substudy 3 will address a mechanism of potential relevance to several behavioral and biological risk factors, omega-3 fatty acids, as studied by observational (Substudy 3a) and experimental methodologies (Substudy 3b – Clinical trial).**

# **The overall sample for the three substudies will include 530 adults (272 for Substudy 3b), 30-54 years of age. Subjects will be scheduled for six (6) laboratory sessions, during which measurements will be obtained on:**

# **functional neuroimaging**

# **ecological momentary assessment of experienced environments**

# **ambulatory blood pressure**

# **neuroendocrine assessments**

# **laboratory clinical evaluations**

# **personality and health behavior assessments**

# **vascular imaging,**

# **molecular genetic analyses**

# Recruiting Letter

This letter is set by the Department of Epidemiology, Recruitment Division.

Recruitment Director: Janet Bonk, 412-383-1564, fax (412-383-1334), e-mail: [jtbonk@imap.pitt.edu](mailto:jtbonk@imap.pitt.edu)

Recruitment Staff: Lizzie

Dear Friend,

You are invited to join the Adult Health and Behavior Project, Phase II (AHAB-II), an exciting and ground-breaking new study sponsored by the National Institutes of Health and the University of Pittsburgh. Every day we read of new scientific findings that claim to show how the many ways that we differ from one another can either increase or decrease our risk for medical conditions, such as heart disease, high blood pressure, or diabetes, later in life. These differences can include weight, cholesterol level, dietary habits, and exercise. Even how much we sleep, our personalities, and the stress we experience in our lives or at work appear to affect our health.

However, the many physical and behavioral differences among people are rarely studied in the same individuals, and not much is known yet about how these factors actually affect health and physical well-being. The aim of the AHAB-II Project is to identify behaviors, genes, and dietary factors that may be important for risk of later heart disease and to examine how differences in personality, diet, stress, exercise, and other common behaviors may affect our bodies. Because we are interested in how genes may also be important for the risk of later heart disease, we will be drawing blood for genetic testing. Your confidentiality will be strictly maintained and your name will never appear on any research materials.

You may be eligible to participate in this study if you meet the following criteria:

- You are in good general physical and mental health.
- You are between 30 and 54 years of age.
- You do not use medications for mental health or cardiovascular problems.
- You are employed at least 25 hours per week outside the home, but do not exclusively work the nightshift.

As a participant you will receive screening for several disease risk factors at no cost to you, including blood pressure, blood glucose, percentage of body fat, diet and physical activity analysis, and full cholesterol profile. If you are eligible and complete the study, you may receive up to $410.00. Parking or transportation and all tests involved in this study are available at no cost to you.

This study is entirely voluntary; you were selected to receive this invitation to participate in AHAB-II by random selection from a publicly available list of persons living in the greater Pittsburgh area. If you want to learn more about the study and discuss your eligibility to participate, please mail our return card or call our staff at our toll free number,

1-800-872-3653. We look forward to hearing from you.

Sincerely,

Stephen B. Manuck Ph.D. Matthew F. Muldoon, MD, MPH

Principal Investigator Co-Investigator

**AHAB 2 Telephone Screening**

**Telephone Screening Script**

Hello, may I speak with _____________________. My name is _____________________ I am calling from the University of Pittsburgh for the Adult Health and Behavior Study-Phase 2 – which we call AHAB-2 research project. I received a card/call that you were possibly interested in participating in the study. Do you have 10 to 15 minutes to discuss your interest in this study?

**If no**: Is there a better time that I could call you back?

**If yes**: AHAB-2 is a study of several hundred men and women from Pittsburgh and surrounding areas, and is sponsored by the National Institutes of Health and the University of Pittsburgh.

The purpose of AHAB-2 is to identify behavioral, physical and genetic differences among people that may be related to their likelihood of developing heart disease later in life.

As you know, people differ greatly in their personalities, in their diets and exercise habits, in the stress they experience at work or elsewhere, as well as in many other characteristics.

In AHAB-2, we are looking at whether normal differences in personality traits, stress at work, genes, and diet affect important heart disease risk factors (such as blood pressure, blood sugar and cholesterol levels) and early signs of heart disease.

In addition, we are studying how stress is experienced in daily life and how personality and diet may be related to certain kinds of brain activity.

If you would like to participate in this study, you would be among 500+ adults between the ages of 30-55.

You would be asked to attend 6 sessions lasting between 2 and 4 hours each.

You would receive $350 for completing all 6 sessions, and would be eligible to receive a $50-$60 bonus. You will also receive information about your own risk factors for heart disease.

You will not be asked to take any medication nor will you be exposed to any x-rays or radiation as part of this study.

Do you think you might be interested in participating in this study? Yes No

Before enrolling you in this study, I need to determine if you are eligible for participation. What I would now like to do is ask you a series of questions about your health to see if you would qualify for a screening visit. Some of the questions are personal and you may find them sensitive, however, you can refuse to answer any question. All information will be kept strictly confidential; your name will never appear on any research materials. Remember, your participation is voluntary.

Do I have permission to ask you these screening questions to determine if you are eligible to participate in this study?

VERBAL CONSENT OBTAINED: Yes No By: ___________ Date: __________

**If no**: Thank you very much for your interest. The information that has been collected about you during this screening call will be destroyed. Thank you and we hope you have a nice day.

**If yes:** Proceed to telephone screening form.

## ELIGIBILITY SCREENING

A. What is your age? ____________

Below 30 or above 56 **🡪 EXCLUSION CRITERION**

30-55

(if 29, consider delayed enrollment, continue screening)

B. Are you of Hispanic descent? Yes No

C. Which of these groups best describes you? (CHECK ONE)

_____ White _____ Asian/Pacific Islander

_____ Black _____ American Indian or Alaskan Native

_____ Other

D. What is your first language? _______________ **IF NOT ENGLISH**, **EXCLUSION CRITERION**

D2. Do you use or have you used English as the primary language spoken in your home for the last 10 years? Yes No **🡪EXCLUSION CRITERION**

E. Are you employed outside the home, that is, do you travel to another location to do your work? Yes No **🡪EXCLUSION CRITERION**

How many hours per week are you at work? ____________ record number

<25 hours per week  **🡪 EXCLUSION CRITERION**

>25 hours per week

E1. How long have you been employed with your company? ____________ record number in years

E1a How long have you been in your current position (if in more than one job, collect duration for the position that involves the largest number of hours per week)?

____________ record number in years

(if less than 1 year, in months)

<1 year  **🡪 EXCLUSION CRITERION**

>1 year

E2. In this current position, how many months have you been working full or part time during the past year?

____________ record number in months

<9 months  **🡪 EXCLUSION CRITERION**

>9 months

*This question is designed for jobs that involve seasonal work, such as landscaping or teaching. We want to make sure that employment involves a commitment of at least 9 months per year in part time or full time work.*

F. In your current position(s), do you ever work the night shift, that is, where your work day starts in the evening and continues after midnight? Yes No

F1. If yes, what hours do you work when you work the night shift? _______________ record hours worked

(e.g., 10 pm – 5 am)

*Interviewer: Night shift = a period of work in which half or more of the hours worked are worked between midnight and 8 am.*

F2. How many times have you worked the night shift over the past year? ____________ record number

If night shift (F2) >12 times over the last year (>once per month, on average), **EXCLUSION CRITERION**

G. Have you completed at least 8 years of school? Yes No **🡪EXCLUSION CRITERION**

H. Are you currently taking any medications for your mental health or your mood? Yes No

*****check medication list for exclusions*****

What medications are you taking? ___________________________________

__________________________________________________________________

I. Are you currently taking any other prescription medications? Yes No

*****check medication list for exclusions*****

What prescription medications are you taking? __________________________

__________________________________________________________________

J. Are you currently taking fish oil, cod liver oil, algae, algal oil or DHA supplements?

Yes **🡪 EXCLUSION CRITERION**

No

Ja. Are you currently taking the fat-blocker Alli?

Yes **🡪 EXCLUSION CRITERION**

No

K. Have you ever had a heart attack, a stroke, bypass surgery or balloon angioplasty?

Yes **🡪 EXCLUSION CRITERION**

No

L. Have you ever been told by a doctor that you had angina?

Yes **🡪 EXCLUSION CRITERION**

No

Ma. Have you ever had a head injury for which you were hospitalized?

Yes No

Mb. Have you ever been knocked unconscious for more than 60 seconds?

Yes No

N. Do you take insulin?

Yes **🡪 EXCLUSION CRITERION**

No

O. Do you take a steroid in pill form, such as cortisone, hydrocortisone, prednisone or deltasone?

Yes **🡪 EXCLUSION CRITERION**

No

P. Do you have asthma or another lung condition?

Yes **🡪** Have you been prescribed or have you used a medication at least 7 days in the past 2 weeks?

Yes **🡪 EXCLUSION CRITERION**

No **🡪** continue to question Q.

No **🡪** continue to question Q.

Q. Have you ever been in an MRI machine before?

Yes No

R. Are you claustrophobic (have a fear of enclosed or confined spaces, such as elevators)?

Yes, definitely **🡪 EXCLUSION CRITERION**

Yes, a little

No

S. Please indicate with a “YES” or “NO” if you have any of the following:

| **YES** | **NO** |  |
| --- | --- | --- |
|  |  | Cardiac pacemaker or internal pacing wires **🡪 EXCLUSION CRITERION** |
|  |  | Electronic or magnetically activated implant or device **🡪 EXCLUSION CRITERION** |
|  |  | Implanted cardioverter defibrillator **🡪 EXCLUSION CRITERION** |
|  |  | Implanted drug device, such as insulin or infusion pump **🡪 EXCLUSION CRITERION** |
|  |  |  |
|  |  | Aneurysm clip, intravascular filter or stent(s) |
|  |  | Any metallic foreign body, (ie BB’s, bullets, pins, screws, rods, plates, wires, shrapnel) |
|  |  | Any other external or internal metallic object, implant or IUD |
|  |  | Any permanent makeup or tatoos |
|  |  | Artificial limb(s), artificial joint replacement |
|  |  | Body art, jewelry or piercing (non-removable) |
|  |  | Metal-post root canal or bridgework, or non-removable braces, or dentures |
|  |  | Embolization coil(s) |
|  |  | Hair extensions, implants or wigs (non-removable) |
|  |  | Non-removable hearing aid(s) or any ear implant |
|  |  | History of EVER having metal in or removed from your eye(s) |
|  |  | History of metal work |
|  |  | Inability to lie flat for an extended period of time, ie one hour or longer |

T. (If female) Are you pregnant or lactating?

Yes **🡪 EXCLUSION CRITERION**

No

U. Do you consume more than 35 alcoholic drinks per week?

Yes **🡪 EXCLUSION CRITERION**

No

If ineligible for AHAB-2, are you willing to be contacted for future research studies? Yes No

V. Do you have any chronic medical conditions? Yes No

If yes, what? _______________________________________(check with study coordinator)

_______________________________________(check with study coordinator)

_______________________________________(check with study coordinator)

W. Do you have any psychiatric conditions? Yes No

If yes, what? _______________________________________(check with study coordinator)

_______________________________________(check with study coordinator)

_______________________________________(check with study coordinator)

AFTER TELEPHONE SCREENING

***IF INELIGIBLE:***  Thank you very much for your interest. Based on these questions, it looks like you are not eligible for AHAB-2. We really appreciate your interest in the study and the time you gave us to answer the questions. The information that has been collected about you during this screening call will be destroyed. Thank you and we hope you have a nice day.

***IF ELIGIBLE:***  Thank you very much for your time. Based on these questions you may be eligible to participate in AHAB-2. Let me tell you a little more about the study.

***IF ELIGIBLE, but further determination is needed (positive response to question R):***  Thank you very much for your time. Based on these questions you may be eligible to participate in AHAB-2, but further determination is needed. Someone from the research center will contact you before your scheduled appointment to discuss the additional information. Let me tell you a little more about the study.

The first step is to come into our clinic to verify your eligibility.

As part of this first visit to our clinic, we will ask you to fast, that is, to have no food or drink except water, after 9 p.m. the night before this first visit.  We are asking you to fast so that we can do a fasting blood draw at this visit.  Of course, the blood draw will not be conducted until after we obtain written consent from you. You should be aware that fasting for some individuals causes mild stomach discomfort, sometimes called, "hunger pains."

This is a 2 to 3 month study consisting of six visits – all located in Oakland. Each visit will last between 2 and 4 hours. During these visits, you will be asked about basic demographic information, medical history, diet, exercise, sleep habits, smoking and alcohol use. A study nurse will draw a sample of blood that will be analyzed for cholesterol, blood sugar, other risk factors of heart disease, as well as genetic factors that may be related to heart disease risk. You will be asked to complete interviews and/or questionnaires about your moods, your personality characteristics, and your work experience. You will be asked to use several monitoring devices for two 2-day periods; undergo a non-invasive and painless brain imaging test to help us understand what parts of the brain are involved in different mental tasks, and undergo a non-invasive and painless scan of your carotid arteries.

At the end of the study, you will receive information about your risk factors for heart disease, including blood pressure, cholesterol and glucose levels, and feedback regarding your diet and exercise. Although some of the procedures cannot be considered part of a clinical evaluation, if a clinically important condition is detected, this information will be made available to you and recommendations for further evaluation will be provided.

We will pay for your parking or public transportation expenses for each visit associated with the study. You will also receive $350 for completing all 6 visits. Do you have any questions at this time? We would like to set up an appointment for you to come into the clinic for an initial screening visit. Would ________(month),

_______(day), at __________(time) work for you?

When you come for your first appointment, please bring all of your prescription medications and supplements that you take with you.

We will send you a packet that contains an appointment reminder, directions to the clinic, and parking/transportation instructions. Please read over the material prior to your appointment. If you have any questions, there will be a contact number included in the packet. Do you have any additional questions? Thank you very much and we hope you have a very nice day!

Eligibility/Exclusion Criteria:

**Eligibility**

The following are eligibility criteria for AHAB-II:

1. Men and women between 30 – 54 years of age.
2. Work at least 25 hours per week
3. English proficiency (English as first language)

# Exclusion

The following are exclusion criteria for AHAB-II:

1. Medical condition:
   1. Heart attack, stroke, bypass surgery
   2. Angina
   3. Severe hypertension (SBP/DBP > 160/100)
   4. Chronic psychiatric or medical condition
   5. Insulin shots for diabetes
   6. Lung disease requiring drug treatment
   7. Liver disease
   8. Kidney disease
   9. Cancer (excluding basal and squamous)
2. Treatment with a disallowed medication
   1. Includes taking fish oil, algae, algal oil or DHA supplements or fat-blocker, Alli
3. Nightshift workers
4. Consumption of more than 35 alcoholic drinks per week
5. Claustrophobia (fear of enclosed or confined spaces)
6. Certain medical devices, implants or other metal objects in or on the body that cannot be removed
7. Tattooed eyeliner
8. Inability to fit into MRI scanner
9. < 8^th^ grade reading skills

## Per Dr. Muldoon e-mail, 11/12/10, eligible for AHAB-II if not taking fish oil supplement for past 4 months.

Eligibility is determined through the telephone screening interview and reviewed at Visit 1 by AHAB Staff through the Eligibility Verification Form.

**MEDICATION CODING**

**Prescription medications**: code all, even those taken infrequently/PRN.

**Non-prescription medications, supplements, vitamins, aspirin, etc.**: code only those taken at least QOD (every other day) over the past two (2) weeks.

**Coding**

0 = Permitted daily or PRN – no restrictions

1 = Disallowed daily or PRN

2 = Disallowed if taken 7 or more days in the past 14 days

# Frequency Coding

Number of days taken in the past 14 days

Last taken coding

1 = today

2 = yesterday

3 = day before yesterday

4 = longer (not taken in past 2 ½ days)

**Visit 1 (3 – 4 hours)**

RESTRICTIONS:

Nothing to eat or drink, except water, after 9 p.m. night before visit. Includes no caffeine 2 hours prior to visit and no alcohol day of visit *(added to protocol 4/2008)*

No tobacco 2 hours prior to visit *(added to protocol 4/2008)*

No exercise 2 hours prior to visit *(added to protocol 4/2008)*

PROTOCOL:

- Ethnicity/Race Tracking Form (NIH purposes)
- Eligibility Verification review
- Informed Consent
- MRI Simulator
- Tanita – body composition
- BP Screening
- Medical History
- Women – Gynecologic History
- Blood draw
  - Lipids, DNA, CPR/IL-6, Fatty Acids, Immune Measures
- Record Structured Interview (SI-TABP)
- Demographic Form *(includes Origin interview, Job characteristics, Ladders, Hollingshead, Family Composition)*
- Tobacco Interview
- Alcohol Use Interview *(moved from V4 to V1 4/2008)*
- Paffenbarger (week and year)
- Food Frequency Interview
- M.I.N.I. *(moved from V3 to V1 4/2008)*
- Sensus
  - ISEL
  - SNI
  - CEQ
  - SSS
  - TCI-NS
  - FES
  - FTND
  - LHA *(added to protocol folder# 2114)*
  - ERQ-2 *(moved from V5 to V1 12/01/2008)*
- 15 hour urine collection instructions/materials

1. **Height**

Have subject remove shoes and stand with heels against the wall and lower top plate so that is gently rests on the top of the head. Record height in **inches** in lab and BMRG folders.

1. **Tanita**

The scale measures percent body fat and resting metabolic rate. Verify that the subject does not have a pacemaker; shoes and socks need to be removed. (pantyhose and tights are OK, need to put distilled water on scale)

***All subjects should be entered as STANDARD***

1. Turn on Tanita body composition analyzer
2. Sanitize weighing platform with Lysol Disinfectant Spray
3. Select pounds for entry of clothes weight
4. Enter 2 or 3 pounds depending on amount of clothing and season.
5. Enter Standard Male or Standard Female
6. Enter age
7. Enter height in feet and inches to the nearest ½ inch
8. Ask participant to stand on scale; wait for results to print out.

Make 2 photocopies of print out: one filed in lab folder; one for subject (along with Tanita explanation)

Record readings and attach original to *Lab Session* form and file photocopy in lab folder (behind *Lab Session form)*

1. Sanitize weighing platform with Lysol Disinfectant Spray

# All subjects are asked: ‘Have you done at least 10 hours of aerobic exercise every week for at least the past 12 months?’

# If positive response, Tanita should be repeated using the *Athletic* setting.

1. **Waist/hip measure**
2. WAIST**:** Measure at the point midway between the costal margin (bottom of the rib cage) and the iliac crest (top of the hip bone).
3. HIPS**:** Measure at the widest point around the greater trochanter (hip joint).

**Obtain measurements from the participant’s side, not from the front.


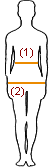


1. **Blood pressure cuff measurement**
   1. Blood pressure measurement procedure is explained to the participant
   2. Using the left arm, measure mid-arm circumference to determine proper cuff size*
   3. Record on blood pressure measurement form.
   4. Participant is asked to remain seated, legs uncrossed, for a period of 5 minutes (set timer) and during blood pressure measurement. Participant may fill out questionnaires or answer minimum benign questions.
   5. Brachial artery is located and the cuff is placed snugly on the left arm: bladder is centered over the brachial artery, 1 -1/1/2 inches above the crease.
   6. Arm is positioned at heart level
   7. Blood pressure should be obtained using the ‘bell’ of the stethoscope.

*Arm circumference measurement: Ask participant flex left arm at a 90 angle and measure the length between the acromiun (shoulder) and olecranon process (elbow) to determine midpoint of arm length. Have the participant relax arm and dangle at side. Place tape measure around determined midpoint of arm to obtain arm circumference. Select cuff size according to chart below.

| **Circumference** | **Cuff size** |
| --- | --- |
| < 23 cm | Small |
| 23 – 30 cm | Regular |
| > 30 – 37.5 cm | Large |
| > 37.5 cm | Thigh |

1. **Medical History**
   1. Have subject complete pages 1 and 2 of Medical History Form (checklist)
   2. Review checklist, document short narrative for any positive responses
   3. Complete Project 2 Medical Hx Measures paperwork
2. **V1 Restrictions**

If participant has not fasted for at least 8 hours reschedule blood draw for Visit 4.

If participant has had caffeine, smoked tobacco or exercised within the past 2 hours, take screening BP and repeat BP toward end of visit.

1. **Blood Pressure**
   1. When timer goes off, determine maximum inflation level (MIL):
      1. Palpate radial pulse and inflate the cuff to approximately 70 mm Hg. Continue to inflate cuff at increments of 10 mm Hg until the pulse disappears to touch. Deflate the cuff.
      2. Remember the level on the manometer when the pulse disappears and add 30 mm Hg at the next BP measurement.
   2. Brachial artery pulse is palpated and stethoscope bell (or diagphram) is placed over the pulse.
   3. Cuff is inflated to MIL.
   4. Cuff is deflated at about 2 mm Hg/sec while listening for first and last K sounds.
   5. Cuff is rapidly deflated after last sound.
   6. Blood pressure reading is recorded.
   7. Take 30 second radial pulse (record as beats per minute)
   8. Steps b – f are repeated for second blood pressure
   9. The cuff is removed from the participant’s arm
2. **Medications**

Record name, dose, frequency, reason taken, and last dose for all prescriptions, mental health/mood meds, OTC meds, vitamins and nutritional/herbal supplements (e.g., St. John’s Wort, Kava Kava, etc)

1. **Rose Questionnaire**

Complete interview and record clinical judgment

1. **Head Injury/birth history**

Complete questions

1. **Gynecological History**

Complete, if applicable. Information from gyne interview should be transferred to green data entry sheet.

1. **Blood Draw Eligibility Questionnaire**

From the blood draw, cholesterol and glucose levels will be determined and provided in a feedback letter after the study has been completed. Portions will be stored for DNA genotyping, fatty acid analysis, immune function analysis and cytokine production.

**Conditions for immune measure ineligibility:**

Do no collect or store samples for CRP/IL6 or collect a green top for the Immune lab if the subject has any of the following medical conditions:

1. **Autoimmune “connective tissue” disorders**. This includes rheumatoid arthritis, lupus, psoriatic arthritis, Sjogren’s syndrome, SICCA syndrome, scleroderma (also called systemic sclerosis), polymyositis/dermatomyositis, mixed connective tissue disease, anklosing spondylitis, polyarteritis nodosa or other types of vasculitis.
2. **HIV/AIDS**
3. **Inflammatory bowel disease (Crohn’s disease and ulcerative colitis)** (“Irritable bowel syndrome” is not included in this group so we should collect the blood samples on those individuals.)
4. **Chronic hepatitis**. This includes hepatitis B and C (not A), autoimmune hepatitis, alpha-1 anti-trypsin deficiency, Wilson’s disease, hemachromatosis
5. **Chronic lung disease**. This includes cystic fibrosis, sarcoidosis, and interstitial lung diseases due to asbestosis, silicosis or radiation. (Bloods should be collected on subjecs with asthma **unless** use inhalers or steroids >/= 7 times in past 14 days).
6. **Chronic Antiviral meds** – Such as Zovirax (acyclovir) for herpes.
7. **Regular use of allergy shots** (draw if given >21 days ago)

If participant is currently taking (or has taken in past 2 weeks) any antibiotics/antivirals OR currently has (or has had in the past 2 weeks) an infection OR currently has (or has had in the past 2 weeks) a cold, flu, or virus (SEVERTIY SCORE >6), reschedule immune function blood draw.

1. **Blood draw**

Lipids

IL-6

CRP

Fatty Acids

DNA

***AHAB-2 BLOOD DRAW PROTOCOL***

AHAB participants will have their blood drawn during the Visit 1 by study nurse. Participants will be asked to abide by the following restrictions:

Nothing to eat or drink, except water, after 9 p.m. night before visit, except water.

The following tubes should be drawn in the order below.

| **Tube & volume** | **Priority** | **Processing** | **Use** |
| --- | --- | --- | --- |
| 10 ml red/grey top  (02-683-98) | 1 | Room temperature, allow 20 min. to clot  *Centrifuge 10 min*  Deliver to Genetics lab | 1.5 ml for lipids, insulin and glucose  plus  3 serum samples @ ≥.9 ml each for storage |
| 10 ml purple top  (02-657-32) | 2 | Room temperature  Deliver to Genetics lab | Buffy coat for DNA extraction  3 EDTA plasma samples @ ≥ .9 ml each for storage |
| 3.15 ml blue top  (22-040-046) | 3 | Remove 1 ml whole blood  Place in lab refrigerator  Deliver to Genetics lab | 1 ml whole blood, stored at -70^o^C  RBC sample for fatty acid analysis.  Store in original vacutainer tube |
| Three 4.5 ml blue tops  (02-688-26) | 4 | Room temperature – 20 min.  *Centrifuge 10 min*; aliquot plasma.  Deliver remainder to Genetics lab. | Plasma: two - .5 ml for IL6 by Marsland lab,  .5 ml for CRP (University of Vermont)  3 citrate storage samples @ ≥.9 ml  Remainder to Genetics lab for buffy coat. |
| 6 ml green top  (02-687-97) | 5 | Room temperature  Deliver to Marsland lab | Whole blood stimulated cytokine production |

Fill all tubes completely.

Record time of blood draw on Blood Draw Eligibility form.

Document any difficulties with blood sampling (this would include tubes that are incompletely filled or are not obtained) and which tubes are successfully drawn on the AHAB-II blood tracking form.

The absolute minimum blood needed for eligibility for the study is 4 ml in the 1^st^ tube (red/grey). If this is the only blood that is successfully drawn, obtain cheek swab sample for DNA extraction.

***Cheek swab protocol***

Collection, Labeling and Delivery

1. Label four (4) swab tubes with either a AHAB2 – Ferrell DNA or AHAB2 – Blue top label***.***
2. Collect 4 swabs, 2 from each cheek

1a. Have subject thoroughly rinse out mouth twice with water

1b. Collect tissue by rolling the swab firmly on the inside of the cheek, approximately 20 times on each side, making certain to move the brush over the entire cheek

1. Air dry swabs for 10-15 minutes at room temperature and place them back into swab tube
2. Deliver double-bagged in biohazard bag at room temperature to **Genetics Lab** before 11:30 AM (324 Parran Hall – Nancy Petro [4-6791]

**Immune Blood Procedures (May 2012)**

**Conditions for immune measure ineligibility:**

Do no collect or store samples for CRP/IL6 or collect a green top for the Immune lab if the subject has any of the following medical conditions:

- **Autoimmune “connective tissue” disorders**. This includes rheumatoid arthritis, lupus, psoriatic arthritis, Sjogren’s syndrome, SICCA syndrome, scleroderma (also called systemic sclerosis), polymyositis/dermatomyositis, mixed connective tissue disease, anklosing spondylitis, polyarteritis nodosa or other types of vasculitis.
- **HIV/AIDS**
- **Inflammatory bowel disease (Crohn’s disease and ulcerative colitis)** (“Irritable bowel syndrome” is not included in this group so we should collect the blood samples on those individuals.)
- **Chronic hepatitis**. This includes hepatitis B and C (not A), autoimmune hepatitis, alpha-1 anti-trypsin deficiency, Wilson’s disease, hemachromatosis
- **Asthma if requiring daily steroids or daily (7 or more times in past 2 weeks) inhaler therapy**. (For subjects not taking daily meds, an asthma diagnosis is noted but immune labs are drawn.)
- **Chronic lung disease (other than asthma)**. This includes cystic fibrosis, sarcoidosis, and interstitial lung diseases due to asbestosis, silicosis or radiation.
- **Chronic Antiviral meds** – Such as Zovirax (acyclovir) for herpes.
- **Chronic glucocorticoid medication (e.g., prednisone) for any indication**
- **Regular use of allergy shots or recent vaccination** (draw if given >21 days ago)
- **Acute infections for which antibiotics or antiviral medication is prescribed (past 2 weeks)**
- **Cold or flu in past 2 weeks if symptom score >5**

Immune Function Blood Draw

Ineligibility Protocol (V1)

1. If answers **YES** to **Question #2**, *taken antibiotics in past 2 we*eks then do not draw Immune Functions (green tube), do not preserve CRP or IL-6 samples from blue tubes.
   1. Verify dates antibiotic to be taken, noting last day dose taken.
   2. Blood may be drawn 2 weeks from last dose. (EXCEPTION IS Z-PACK – add 5 (five) extra days).
   3. Place RESCHEDULE Slip on front of tracking with date filled out.
2. If answers **YES** to **Question #3**, *currently have or have had infection in past 2 weeks*, verify type of infection and if treated.
   1. Determine when participant will be infection free based on treatment or type of infection
   2. Blood may be drawn 2 weeks from date participant is infection-free.
   3. Place RESCHEDULE SLIP on front of tracking with date filled out.
   4. Send slip to Yao lab that CRP will follow (See below).
3. If answers **YES** TO **QUESTION #4,** *currently have or have had cold or flu in past 2 weeks,* rate symptoms as per Blood Draw Eligibility Questionnaire guidelines.
   1. If < 6 (six) on day of appointment Blood may be drawn 2 weeks from that date.
   2. If > 6 (six) screen at next appointment for eligibility for blood draw.
   3. Blood may be drawn 2 weeks from score < 6 (or if no illness in past 2 weeks for part b.
   4. Place RESCHEDULE Slip on front of tracking with date filled out.
4. If **blood not drawn at V1**:
   1. Explain reason blood not drawn and ask if okay to draw fasting labs for immune functions at future visit. If okay:
      1. Place green tube and unattached label in clear plastic bag
      2. Place a single blue tube in bag and mark DISCARD AFTER PIPETTE on label
      3. Place CRP and IL-6 labels on serum tubes and place in bag with green tube
      4. Attach to new Blood Draw Eligibility Questionnaire and place in Lab Folder.
5. Scheduler will schedule a Blood draw with V4 per Reschedule Slip
   1. Fasting blood draw to be scheduled in am.
   2. Leave Reschedule slip on front of chart
   3. Note in tracking folder beside appointment with scheduled blood draw to give reminder that draw is scheduled and to follow same restrictions as regarding smoking, caffeine and exercise as at V1.
6. Reminder call to include reminder to fast for blood draw and follow same restrictions regarding smoking, caffeine and exercise as at V1.
7. Follow-up Immune Blood draw
   1. Fill out Blood Draw Eligibility Questionnaire
   2. If eligible, do blood draw and process as per protocol. Discard blue tube after serum transfer of CRP and IL-6.
   3. Transfer Question #6 *Have you received any vaccinations or shots in past month* to Health History
8. **15-hr urine collection instructions**

This collection evaluates the total amount of urine your body makes over a 15-hour period of time.

- 1. Prepare the urine container:
     1. Empty tube of sodium metabisulfite into the container (wear latex gloves while handling test tube).
     2. Discard test tube
     3. Secure lid on the urine container
  2. *The container contains a preservative, inform subject to follow these guidelines:*
     1. Keep the container upright at all times
     2. Avoid touching the lip of the container
     3. Do not allow your skin to come in contact with the liquid
  3. Container should be refrigerated at all times (starting when participant leaves the office, until used, and after collection is completed)
  4. On the day of urine collection, the first time you urinate after 5:00 pm, ***flush the urine down the toilet***. Record date and time on lid of the urine container on the **“start”** lines. This determines the time the collection begins, but the bottle should still be empty.
  5. Every time you empty your bladder over the next 15-hours, urinate directly into the container
  6. Keep the container closed tightly and refrigerated once the collection has begun
  7. As close as possible to the end of the 15-hour period, empty bladder into container and record date and time on the lid of the urine container on the **“finish”** line.
  8. Return to the BMRG at Visit 2.

1. **SI-TABP**

This is a structured interview, meaning that every participant in the AHAB study will be asked the same questions and not all of the questions will apply to everyone. I am going to record this interview so that this interview can be coded at a later day.

Record for coding using digital recorder; files will be coded at a later date by all RAs.

Save using AHAB ID number

Upload to server folder (T:\Interviews)

1. **Demographics**

Subject should self-report pages 1 – 8. Review and ask follow-up questions, if necessary.

Sections B thru F should be completed as an interview.

Demographic data should be transferred onto green data sheets**.**

1. **Tobacco Use Interview**

Complete as an interview.

Tobacco data should be transferred onto green data sheets**.**

1. **Alcohol Use Interview**

Complete as an interview.

Alcohol data should be transferred onto green data sheets**.**

1. **Paffenbarger**

We are interested in physical activity over the past (week/year):

Question 1 asks about how many blocks (12 blocks=1 mile) walked on average, each day. We are interested in walking done in the daily routine that maybe take for granted, for example walking to the bus stop or from desk to the restroom, etc. Do not include walking done specifically for exercise; that should be indicated on the bottom of the page under sport activities.

Question three asks about how many stairs climbed UP each day (include flights UP only).

The bottom of the page asks about sport activities. Here we are interested in any activities done once per week [or four or more times per year] These activities do not necessarily have to be organized sports, such as soccer or football, they can be things like dancing, biking, or swimming, just as long as they are done at least once a week [or four or more times per year].

1. **Food Frequency Interview**

<http://www.nutritionquest.com/login/>

user name: subject ID

password: ****

group ID: 268

Upon completion of interview, print copy of report for participant.

Complete question 3 of Fish Oil Eligibility (FFQ: DHA and EPA (combined) < 0.3 grams)

1. **M.I.N.I.**

Assesses major psychiatric disorders in DSM-IV

If subject endorses suicidal ideation during the Mini interview, contact:

1. Dr. Anna Marsland @ 412-370-5622. (cell)
2. If Dr. Marsland is not available, please contact Dr. Tom Kamarck @ 412-760-3322 (cell)
3. In the rare case that the research associate feels that the participant poses a potential risk to self or others and neither Dr. Kamarck or Dr. Marsland are available, Campus Police can be called for assistance (4-2121). This is only to be done in the event of an emergency.
4. Participants may also be accompanied to the WPIC Emergency Room (DEC) or can contact WPIC @ 412-624-2000 for further information.

If subject endorses an Axis 1 diagnosis during the Mini interview, provide a referral list (below) and document the event on the referral form (below).

1. **Sensus**

Questionnaires are completed on the three (3) computers located in room 507. Start menu, Programs, Sensus, Run Sensus Q&A 2.0. Choose v1.qar from main menu; click ‘start’, enter subject ID (AHAB ID). Record ID number, date and type of questionnaire on appropriate computer log sheet.

If subject is unable to finish the Sensus questionnaires, he or she can stop at the instruction page of any of the individual questionnaires. Each instruction page has a button labeled quit. Click on the Quit button and make a note in the protocol folder and in the Sensus Mid folder hanging on the wall by 507A.

1. **Eligibility Verification**

Complete top-portion from data that has been collected

Demographics: age, employment, night shift, reading level

RA judgment: language

Alcohol: weekly alcohol consumption

**Visit 2 (3 ½ hours)**

BMRG will contact participant to remind them of the urine collection that starts at 5pm the evening before the V2.

**RESTRICTIONS:**

Abstain from using tobacco and caffeine, eating meals and engaging in exercise for 2 hours prior to visit.

Refrain from alcohol starting at 9 p.m. the night before visit.

PROTOCOL:

- BP assessment
- Urine collection return/urine collection questionnaire *(added to protocol 5/2008)*
- Ambulatory monitoring
  - BP
  - Electronic Diary
- SenseWear
- Actiwatch
- Saliva collection instructions/materials
- 15 hour urine collection instructions/materials
- Follow-up phone calls
- Schedule Visit 3

Between Visits 2 and 3, participants will undergo two 2-day periods of ambulatory monitoring during waking hours, typically on Friday and Saturday and the following Monday and Tuesday, or during comparable periods that include three work days and one nonworking day. During these two monitoring periods, ambulatory blood pressure, electronic diary self-report assessments, and salivary cortisol will be assessed periodically throughout the day. Electronic diary questions will be used to assess mood, social interactions, and other indices of daily psychosocial demand. These questions will also be used to assess smoking, alcohol, and dietary intake. Activity levels during the daytime and sleep quality at night will be assessed using accelerometry devices worn on the upper arm and the wrist. During the evening

prior to Visit 2 and, again, during the evening prior to Visit 3, subjects will collect urine output for a 15 hour period for assessment of urinary catecholamines.

A Research Associate from the BMRG will make five (5) phone calls to participants during the monitoring periods to address equipment concerns and review instructions for all procedures. Phone calls will last no longer than 30 minutes.

**Visit 3 (2.5 hours)**

RESTRICTIONS:

None.

PROTOCOL:

- BP assessment
- Urine collection #2 return
- Return equipment
  - BP monitor
  - Electronic Diary
  - Actical
  - Actiwatch
- Saliva collection return
- Questionnaires
  - JCQ
  - NIOSH QWL
  - Total Workload Scale
  - Work History Questionnaire
  - PERI
  - PSS
  - DAS
  - Handedness *(added to protocol 4/2008)*
  - 7-day Physical Activity Questionnaire *(added to protocol 8/2009)*
- Schedule Visit 4 (if needed)
- Schedule Visit 5 (if needed)

**Visit 4 (3 – 4 hours)**

RESTRICTIONS:

None.

PROTOCOL:

- DDT
- DPX
- IGT
- WASI *(moved to V6 4/2009)*
- STQ *(added to protocol 4/2008)*
- PSQI *(moved from V1 to V6 4/2008) (moved from V6 to V4 4/2009)*
- Life Events List *(moved from V1 to V6 4/2008) (moved from V6 to V4 4/2009)*
- Places Lived Questionnaire *(moved from V1 to V6 4/2008) (moved from V6 to V4 4/2009)*
- Sensus
  - NEO
  - PANAS-X
  - BIS
  - MES
  - SPAQ *(added to protocol 4/2008)*
  - Mach IV *(added to protocol 10/3/2008)*
  - ZTPI *(added to protocol 10/3/2008)*
  - IRI-EC *(added to protocol 10/23/2009)*
  - AATQ *(added to protocol 03/11/2010)*
- Significant Other Forms

NEO S/F and PANAS S/F – given to subject, to be completed by spouse/partner, friend, significant other and returned in postage-paid envelope

- Supplementation introduction
  - Eligible
  - Interested
- Schedule Visit 5 (if needed)
- Schedule URL (if needed)

1. **DDT** (Time discounting task - computer administered)

CLICK ON *DDTA* SHORTCUT ON SCREEN

SELECT ID FROM TOP TOOLBAR THEN ENTER SUBJECT AHAB- II ID

SELECT START TO BEGIN

*During this task, you will be presented with a series of choices. In each case, you will be asked to choose between a variable amount of money available NOW, and a fixed amount of money available after a delay. For each choice, the dollar amounts and delays will first be presented on the screen in white. During this time, no response will be counted. After 2 seconds, the choices will appear in black, and you will then be able to make your selection. Please consider each choice carefully, and answer the questions as if it were real money. Some questions may be repeated. Please press any key when you are ready to begin.*

2. **DPX** (computer administered)

Follow instructions on hand-out. Using the example “TARGET V. NON-TARGET” card to illustrate the task.

*DPX Task (Dot Pattern Expectancy)*

Access through desktop shortcut

When prompted enter value of 4 for session number followed by participant AHAB-II ID number

I. Instructions:

*During this task you will be asked to press one of these two buttons after each of these patterns (show pattern card to participant).*

*You will press button* ***N (non-target)*** *after all dot patterns you see except this pattern. However, you will only press button* ***T (target)*** *after this dot pattern when it directly follows this dot pattern. If you are shown this dot pattern following any other dot pattern, then you will press* ***N*** ***(non-target)****.*

*Clarify by explaining to participant something similar to the following:*

*The only time you should press button* ***T (target)*** *is when you are shown this dot pattern directly after being shown this dot pattern.*

*Remind participant to respond as quickly yet accurately as they can.*

3. **IGT** (computer administered)

Accesses through desktop shortcut then complete the following steps:

1. Select *“New Client File”*
2. Enter the following:
   - *AHAB-II ID*
   - *DOB*
   - *Sex*
   - *Years education completed*
3. Select tab to right, “”, to enter *Ethnicity*
4. Once above info entered, select *“Add Protocol”*

Read the following to participant (administration instructions):

*In front of you on the screen, there are 4 decks of cards A, B, C, and D. I want you to select one card at a time, by clicking on the card, from any deck you choose. Each time you select a card, the computer will tell you that you won some money. I don’t know how much money you will win. You will find out as we go along. Every time you win, the green bar gets bigger. Every so often, however, when you click on a card, the computer tells you that you won some money, but then it says that you lost some money too. I don’t know when you will lose, or how much you will lose. You will find out as we go along. Every time you lose, the green bar gets smaller. You are absolutely free to switch from one deck to the other at any time, and as often as you wish. The goal of the game is to win as much money as possible, and if you can’t win, avoid losing money as much as possible. You won’t know when the game will end. You must keep on playing until the computer stops. I am going to give you this $2000 credit, the green bar, to start the game. The red bar here is a reminder of how much money you borrowed to play the game, and how much money you have to pay back before we can see how much you won or lost. It is important to know that just like in a real card game, the computer does not change the order of the cards after the game starts. You may not be able to figure out exactly when you will lose money, but the game is fair. The computer does not make you lose money at random, or make you lose money based on the last card you picked. Also, each deck contains an equal number of cards of each color, so the color of the cards does not tell you which decks are better in this game. So you must not try to figure out what the computer is doing. All I can say is that some decks are worse than the others. You may find all of them bad, but some are worse than the others. No matter how much you find yourself losing, you can still win if you stay away from the worst decks. Please treat the play money in this game as real money, and any decision on what to do with it should be made as if you were using your own money.*

Upon completion of task:

1. Press ***Esc*** key to exit
2. Enter password at prompt (this will exit the program)
3. ***save changes***

4. **PSQI**

This questionnaire asks about sleep quality over the past month. Question 10 asks about a bed partner or roommate; answer based on what your partner or roommate has reported over the past month,

5. Places Lived Questionnaire

This questionnaire looks at where the subject lived during childhood; questions about the house or the apartment lived in at age 5, 10 and 15.

6. Life Events List

After subject completes this questionnaire, check that all questions have been answered - also check for discrepancies, i.e., subject checked no for whether the event happened, but then rated the severity of the event, etc.

7. **NEO S/F and PANAS S/F**

*I’m going to give you a couple of questionnaires to take home for a friend or family member to complete.* (Show questionnaires). *Your significant other is asked to answer several questions about you and return it to us in a postage-paid envelope. They don’t have to provide any information about themselves to us other than their relation to you. Are there two people that know you well and would be willing to answer some questions about you? The best people are a spouse or partner; a parent, brother or sister, or child. If you’ll tell me their names, I’ll prepare a letter that will explain to your friend or family member what we’re asking them to do.*

Complete Significant Other form, record:

gender

relation

how well the subject knows the individual that will complete the form

If the subject does not know two people well enough, send one (or none). The questionnaires can be mailed to a parent or other family member who lives out of the area - we can pay postage for mailing the questionnaires. Get envelopes ready while subject is completing questionnaires and give them to subject before they leave for the day.

8. **Sensus** (NEO-PI-R, PANAS-X, BIS, MES, SPAQ, Mach IV, ZTPI, IRI-EC, AATQ)

Questionnaires are completed on the three (3) computers located in room 507. Start menu, Programs, Sensus, Run Sensus Q&A 2.0. Choose v1.qar from main menu; click ‘start’, enter subject ID (AHAB ID). Record ID number, date and type of questionnaire on appropriate computer log sheet.

If subject is unable to finish the Sensus questionnaires, he or she can stop at the instruction page of any of the individual questionnaires. Each instruction page has a button labeled quit. Click on the Quit button and make a note in the protocol folder and in the Sensus Mid folder hanging on the wall by 507A.

9. **Fish Oil Supplementation Introduction**

Research suggests Fish Oil in food or supplements may:

- Reduce our risk of developing heart disease
- May improve our memory and mood

**Our study**:

- Recruiting 272 participants
- Randomized study – half given fish oil and other half of participants given placebo containing vegetable oil
- Participants and nurse are blinded regarding randomization
- Take 2 capsules daily for 4 months (**Show pill packet to participant**)
- At the end of the study you will complete questionnaires and interviews to measure mood, memory and related characteristics for comparison to AHAB II measures
- We will repeat blood pressure, heart rate, and blood tests also.
- Payment - $225. An additional $50 if you complete 70-79% of the electronic diary questions **or** an additional $100 if you complete 80% or more of the electronic diary questions

**Review Time Line**

- Primary side effect is belching or fishy taste when taking fish oil. A “fresh minty taste” has been added to reduce this side effect. Side effects will be discussed in detail prior to signing consent and questions will be answered then.
- This age group is not used to taking medications so we will offer tips for adherence and developing habits for taking the fish oil.
- They will not wear the blood pressure monitoring devices during this study.
- Visit 1 and Visit 2 will also entail scheduling future appointments.

**Send consent home with participant for review if interested.

**Visit 5 (2 hours)**

RESTRICTIONS:

Abstain from caffeine, tobacco products and exercise for 3 hours prior to appointment.

Refrain from drinking alcohol and taking non-essential medications for 12 hours prior to appointment.

Participants will be asked about any non-removable medical devices or implants, and about any other sources of metal in or on their bodies. In cases of suspected prior exposures or implants that would pose a risk for magnetic resonance imaging, such participants will receive a plain film x-ray exam prior to the MRI study. This x-ray exam will determine the amount and location of any metal within the participant’s body, and thus, whether the participant can be safely scanned in the MRI.

PROTOCOL:

- Screening for contraindications of MRI scans
  - Metallic objects
  - Pregnancy
- ARP
- VSRP
- fMRI – Emotion Regulation *(exploratory component added 11/2008; added for all subjects 10/23/09)*
- ERQ Post-Task Questionnaire *(exploratory component added 11/2008; added for all subjects 10/23/09)*
- ERQ *(moved to V1 12/01/2008)*

Exploratory MRI component and ERQ Post-Task Questionnaire added for 50 subjects *(November, 2008)*

Exploratory MRI component and ERQ Post-Task Questionnaire added for all subjects *(October 23, 2009)*

**Visit 6a (4 hours)**

RESTRICTIONS:

Abstain from using tobacco and caffeine, eating meals and engaging in exercise for 2 hours prior to visit. *(added to protocol 5/2008)*

Refrain from drinking alcohol the day of this visit. *(added to protocol 5/2008)*

PROTOCOL:

- HRV
- Endothelial Function *(added to protocol4/2009; removed from protocol 6/16/10 – no subjects completed )*
- PSQI *(moved from V1 to V6 4/2008) (moved to V4 4/2009)*
- Paffenbarger – week
- Life Events List *(moved from V1 to V6 4/2008) (moved to V4 4/2009)*
- Places Lived Questionnaire *(moved from V1 to V6 4/2008) (moved to V4 4/2009)*
- Neuropsych (spatial span, digit span, 4-word memory test, Rey auditory learning, digit vigilance, trail making, Stroop) added to protocol May, 2008; folder #2053)
- WASI *(moved from V4 to V6 4/2009)*
- Dot Probe Task *(added to protocol 5/2009)*
- BDI
- Sensus
  - CESD
  - STAI
  - CMHI
  - STAXI
  - BPAQ
  - HS
  - MCSD
  - LOTR
  - SRA *(added to protocol 10/3/2008)*
  - MAAS *(added to protocol 12/01/2008)*
  - QAQ *(added to protocol 5/2009)*
- Payment

**HRV (RSA)**

Setup computers for data collection

1. On Gateway Computer, login

-username = AHAB

-password = testing

1. Double click “ACQ 8.0” Icon to open MindWare
2. Click “Start”
3. Click “Acquire data”
4. Select “HRV Data” Folder
5. At prompt “Please enter a filename”, enter “AHABIDur”
6. Hit “Enter” or click “OK”
7. Switch to Mr. Data Computer using KVM switch
8. Mr. Data monitor should be set on “Reactivity” (default), hit “Enter”
   1. At C:\AHAB prompt type “ahab&”, then hit “Enter”
   2. Enter subject’s “AHAB ID”, then hit “Enter”
   3. Select “1” (RSA), then hit “Enter”
   4. Hit “Enter” again
   5. Check to make sure settings at the bottom of the screen are correct (blue print at bottom of screen)
      - You should NOT need to change anything
      - Rate = 1 Khz
      - a(5-digit ID)ur
      - #Channel = 2
      - Test time = 300 (seconds)
      - Rest time = 0
      - Cycle time doesn’t matter, it can be anything
9. Switch to Gateway Computer using KVM switch

Computers are now ready for data collection.

*“Do you need to use the restroom before we get started in the lab?”*

*“Do you have a cell phone or pager on you today? We’re going to ask that you turn it off while we’re in the lab today because it may interfere with some of the monitoring devices that we use.” (Pt. can leave phone with RA while in Chamber 2 if they refuse to turn off device).*

ECG Overview & Preparation

Lead pt. into chamber

*-Ask medication questions & segue into ECG explanation.*

*“We will be performing an ECG or electrocardiogram. This will record electrical signals from your heart. The ECG is a simple non-invasive procedure. I will simply place three sensors on the surface of your skin, one on each wrist and one on your left ankle. During this procedure you will sit still with this respiration belt around your waist and your arms & legs will be still with your palms facing up. I will need to rub your wrists and ankle with some alcohol, and then the sensors will go on just like Band-Aids.”*

*“If you will, please stand for me so that I can put this respiration belt around your waist. It will measure your breathing.”*

- Place respiration belt around abdomen so that it fits snugly while subject is seated
- Confirm from subject that belt is snug but not too tight when seated
- Squeeze bulb 1or 2 times (may need to be adjusted if respiration signal is small)

*We will begin with a five-minute resting period. During this time, I will need you to sit as still and as quiet as possible. After the first five minutes, there will be another five-minute resting period during which I will again ask you to be as still and as quiet as possible. However, for the second five-minute period, I will ask you to breathe according to a regular pattern. I’ll explain this to you in more detail before we begin that portion of the session.”*

*“Do you have any questions so far?”*

- Put gloves on
- Rub wrists & left ankle with alcohol and gauze and attach wrist & ankle sensors for ECG

Sensors should be attached as follows:

*RA* = *Right Wrist*

*LA* = *Left Wrist*

*LL* = *Left Ankle (ISO Ground)*

RSA Breathing Tasks

*“We are now ready to begin the first five-minute rest period. Remember to sit as still and as quietly as you can. Go ahead and move or adjust to get comfortable, but be careful not to pull any of the wires or belt loose. Let me know when you are comfortable so that I can check the signals on the computer. I will be right outside the door during the measurements and there is a microphone in this room in case you need anything.”*

- Once subject indicates s/he is comfortable, check respiration signal on MindWare (Gateway) by clicking “Start”—the graph should look like a sine wave (note if wave is shallow, it may be difficult to score later so you’ll want to be sure that you can clearly see breathing motions)
- Adjust belt or bioamplifier as needed to obtain visible respiration signal
- Click “Stop” on MindWare (Gateway)
- Click “Exit”
- Click “Acquire Data”
- Select “HRV Data” Folder
- Select file with “AHABIDur”
- Computer will ask if you want to replace the file…Select “Replace”
- Switch to Mr. Data Computer

*“The signals look good so we can start. Again, just sit still and breathe as you normally would. Please don’t talk and just do your best to stay awake. I’ll let you know when we’re finished. Ready?…Begin”*

- On Mr. Data keyboard hit F1
- Switch to Gateway
- Click “Start” on MindWare (Gateway)
- Mr. Data will stop collection after 5 minutes
- Click “Stop” on MindWare (Gateway)

*“Okay, we’re finished with the first five-minute rest period. Just give me one minute to setup the computer and I’ll be in to give you instructions for the second rest period.”*

- Click “Exit” on MindWare
- Click “Acquire Data”
- Select “HRV Data” Folder
- Type “AHABIDpr”
- Switch to Mr. Data computer
- Walk around to where subject is to give instruction for paced respiration

*“We are now ready to move onto the second rest period. This time I would like you to breathe according to a regular pattern. To help you do that, the computer will generate two different sets of tones over and over again. The first set of tones is higher in pitch and during this set I would like you to inhale as smoothly and evenly as you can. Then, during the lower pitch set, I would like you to exhale as smoothly and evenly as you can. You should be aware that the sets of tones are not equal in length, the inhale set has four beeps and the exhale set has five. Remember in for four, out for five. It is not necessary that you take deep breaths, just so you stay on pace. Again, I ask that you remain as still as possible. I will give you a few moments to practice and get used to the tones. Then, once I see that you are on pace, I will reset the computer and we will start the task. Any questions?”*

- If subject has no questions, return to computers and hit “Enter” on Mr. Data keyboard
- Switch to Gateway computer
- Click “Start” on MindWare
- Check to see if breaths are even (sine wave should be virtually symmetrical)
- If waves are lopsided, have subject adjust accordingly
- Once subject is able to produce 3 cycles of symmetrical waves you can begin the task
- Click “Stop” on MindWare
- Click “Exit”
- Click “Acquire Data”
- Select “HRV Data” Folder
- Select File with “AHABIDpr”
- Replace file?...Select “Replace”
- Switch to Mr. Data
- Hit “Enter” on Mr. Data

*“It looks as though you have the hang of it so we can get started. Are you comfortable? (allow subject to adjust if necessary). Okay, once I say begin, the computer will delay for a second, then it will start with an inhale set, followed by an exhale set at the same rate that you just practiced. This task will last 5 minutes. Again, please don’t move or talk and do your best to stay awake. Try to stay on pace and breathe as smooth and as evenly as possible. Ready?…Begin”*

- Hit “F1” on Mr. Data
- Switch to Gateway computer
- Click “Start” on MindWare
- Monitor MindWare closely to be sure subject stays on pace

1. If subject cannot achieve pace after 10 seconds, verbalize when to inhale and exhale by saying “in” and “out” for next three sets
2. If subject remains on pace for at least 90 consecutive seconds task is complete
3. If subject cannot stay on pace after first two minutes start task again (refer to trouble shooting guide for instructions on how to reset the task)

- Mr. Data will stop collection after five minutes
- Hit “2” (exit), then “Enter” on Mr. Data
- Click “Stop” on MindWare

*“Okay, we are finished with the ECG and we can remove the equipment now.”*

- Remove ECG leads and hang on wall
- Allow participant to remove adhesive sensors from wrists
- Have subject stand up and loosen valve on respiration belt, then remove

*Note: If you are running the last ECG of the day, please change the battery.*

2. **Paffenbarger - week**

We are interested in physical activity over the past (week):

Question 1 asks about how many blocks (12 blocks=1 mile) walked on average, each day. We are interested in walking done in the daily routine that may be taken for granted, for example walking to the bus stop or from desk to the restroom, etc. Do not include walking done specifically for exercise; that should be indicated on the bottom of the page under sport activities.

Question three asks about how many stairs climbed UP each day (include flights UP only).

The bottom of the page asks about sport activities. Here we are interested in any activities done once per week. These activities do not necessarily have to be organized sports, such as soccer or football, they can be things like dancing, biking, or swimming, just as long as they are done at least once a week.

3. BDI

If subject scores >0 on the BDI suicide question (#9), contact:

1. Dr. Anna Marsland @ 412-370-5622. (cell)
2. If Dr. Marsland is not available, please contact Dr. Tom Kamarck @ 412-760-3322 (cell)
3. In the rare case that the research associate feels that the participant poses a potential risk to self or others and neither Dr. Kamarck or Dr. Marsland are available, Campus Police can be called for assistance (4-2121). This is only to be done in the event of an emergency.
4. Participants may also be accompanied to the WPIC Emergency Room (DEC) or can contact WPIC @ 412-624-2000 for further information.

If subject scores > 16 on BDI, provide a referral list (below) and document the event on the referral form (below).

4. Dot Probe task

Please close blinds and turn off any lights to make room as dark as possible.

Ask subject if they are right or left handed and mark on the tracking sheet.

Subjects are instructed to use their left index finger to press “Z” and their right index finger to press “M”.

1. Administer 1st practice task (“DP Prac 1” shortcut on the desktop):
   - Enter subject ID
   - Subject will read directions on screen; they can press any key to advance until final screen. At that point, tester will advance to task by clicking mouse only when subject is ready.
   - Subjects are asked to look at fixation cross at center of screen at all times. Pairs of SHAPES will appear at regular intervals, and they are asked to press either Z or M as quickly as possible when a blue dot appears (they press **“Z” if dot is on left** and **“M” if dot is on right**).
   - Emphasize that **SPEED** is essential - subject is encouraged to click either Z or M as fast as they can.
   - Practice is approximately 1 minute long
2. Administer 2nd practice task (“DP Prac 2” shortcut on the desktop):
   - Same directions as above
   - Subjects are asked to look at fixation cross at center of screen at all times. Pairs of FACES will appear at regular intervals, and they are asked to press either Z or M as quickly as possible when blue dot appears (they press **“Z” if dot is on left** and **“M” if dot is on right**).
   - Emphasize that **SPEED** is essential - subject is encouraged to click either Z or M as fast as they can.
   - Practice is approximately 1 minute long
3. Administer dot-probe task (“DP” shortcut on the desktop):
   - Enter subject ID
   - Same directions as in practice task
   - Again, emphasize speed, and also let subject know you can’t talk with them during the task.
   - Task is approximately 10-11 minutes long.
4. **DO NOT** save changes to task (if this dialogue box appears at end of task, click “no”) – the program will automatically be saving the data.

5. **Neuropsych**

**Spatial Span**

Administration: (WMS Stimulus Booklet 1)

*Forward*:

-Place Spatial Span Board on testing table.

-Directions: *“Now I want you to do exactly what I do. Touch the blocks I touch, in the same order.”*

-Touch blocks with pen/pencil at rate of one block per second. Record the order in which participant taps blocks on Spatial Span administration sheet.

-Adhere to discontinuation criterion on Spatial Span administration sheet if applicable.

-Allow participant to change answer up to the start of the next trial.

*Backward*:

-Directions: *“Now I am going to touch some more blocks. This time when I stop, I want you to touch the blocks backward, in the reverse order of mine. For example, if I touch this block* (Cube 3)*, then this one* (Cube 5)*, what would you do?”*

-If participant responds correctly, say *“That’s right”* and remind participant to touch the blocks in reverse order, then move on to the first trial of Item 1.

-If participant responds incorrectly, examiner should say *“No, I touched this one, then this one;…”* and instruct participant how to do the subtest correctly as per directions in the Stimulus Booklet 1 Spatial Span Backward section.

-Touch blocks with pen/pencil at rate of one block per second. Record the order in which participant taps blocks on Spatial Span administration sheet.

-Adhere to discontinuation criterion on Spatial Span administration sheet if applicable.

-Allow participant to change answer up to the start of the next trial.

Scoring:

One point is awarded on Spatial Span administration sheet for every trial of every item that is correct. The total points are then tallied and recorded on administration sheet under “Forward Total Score” and “Backward Total Score” and “Total Score”.

Scores should be transferred to Green Data Sheet (Neuropsych Scoring – V6) under Item 1 (Spatial Span). Age-scaled scores should be derived from Table D.1 of the WMS –III Administration and Scoring Manual (p. 139-141) and should be entered on the Green Sheet.

**Digit Span**

Administration: (WMS Stimulus Booklet 1)

*Forward*:

-Directions: *“I am going to say some numbers. Listen carefully, and when I am through, I want you to say them right after me. Just say what I say.”*

-Read digits at rate of one per second. WMS administration booklet instructs examiner to “drop your voice inflection slightly on the last digit in the sequence.”

-Record order in which participant says numbers on Digit Span administration sheet.

-Adhere to discontinuation criterion on Digit Span administration sheet if applicable.

-Allow participant to change answer up to the start of the next trial.

*Backward*:

-Directions: *“Now I am going to say some more numbers. But this time when I stop, I want you to say them backward. For example, if I say 7-1-9, what would you say?”*

-If participant responds correctly, say *“That’s right”* and move on to the first trial of Item 1.

-If participant responds incorrectly, examiner should say *“No, you would say 9-1-7…”* and instruct participant how to do the subtest correctly as per directions in the Stimulus Booklet 1 Digits Backward section.

-Read digits at rate of one per second.

-Record order in which participant says numbers on Digit Span administration sheet.

-Adhere to discontinuation criterion on Digit Span administration sheet if applicable.

-Allow participant to change answer up to the start of the next trial.

Scoring:

One point is awarded on Digit Span administration sheet for every trial of every item that is correct. The total points are then tallied and recorded on the administration sheet under “Forward Total Score” and “Backward Total Score” and “Total Score”.

Scores should be transferred to Green Data Sheet (Neuropsych Scoring – V6) under Item 2 (Digit Span). Age-scaled scores should be derived from Table D.3 of the WMS –III Administration and Scoring Manual (p. 172-177) and should be entered on the Green Sheet.

**Short-term Memory Test (Four-word Memory Test)**

Administration: (Directions are located on every set of administration sheets)

Directions: *“’I’m going to read you four words, which I would like you to try to remember. In order to make your task more difficult, however, after I read the fourth word, I’m going to read a 3-digit number, like 100. As soon as I read you that number, I want you to begin counting backwards by threes as rapidly and as accurately as you can. I want you to continue doing that until I tell you to stop. At that point you’ll tell me what the four words are.’*

*‘How good are you at counting backwards by threes? Let’s try it. Start from 100.’ [provide practice – let subject count to approximately 70]*

*‘Before we begin, I want to quickly review what you’ll be doing. First, you’ll hear 4 words – and I want you to try to remember those. Then you’ll hear a number – and I want you to count backwards from it by threes. After a while, I’ll tell you to stop, and you’ll tell me the words.’*

*‘Here are the first four words I want you to try to remember…’”*

-Read words at rate of one per second.

-Upon participant recall after examiner says “Stop,” circle correct words and notate the order in which they are said.

-Draw a line through words that are not said by participant.

-Write incorrect words in the space provided for “incorrect response” on administration sheet.

-There is no discontinue rule, so all items should be administered.

Scoring:

-Tally all correct words (circled) for each of the three times and record the number of correct words in the table on the bottom of the second administration sheet in the column labeled (#) for 5 seconds, 15 seconds, and 30 seconds respectively. Add these items to determine the total number of correct words and record this number on the administration sheet as well.

-Tally all incorrect words that are written in the “incorrect response” column for each of the three times and record these numbers in the table on the bottom of the second administration sheet in the column labeled (I) for 5 seconds, 15 seconds, and 30 seconds respectively. Then, add the (I) items to determine the total number of incorrect responses and record this number in the table.

-Disregard columns (P) and (E) of the table.

-Scores should be transferred to the Green Sheet (Neuropsych Scoring – V6) under Item 3 (Four-word Memory Test).

**Rey Auditory Verbal Learning Test**

Administration: (Directions are located on every set of administration sheets)

-Directions (Recall A1): *“I am going to read a list of words. Listen carefully, because after I stop, I want you to say back to me as many words as you can remember. It doesn’t matter in what order you repeat them. Just try to remember as many as you can.”*

-Words should be read at rate of one per second. When examiner has finished reading words, participant should be asked to say as many words as he can remember.

-Participant responses should be recorded in order on the administration sheet in the column labeled (Recall A1). A simple number in column (Recall A1) next to the word is sufficient. For example, if the participant says “Moon, Hat, Turkey” then the examiner should place a 1 next to the word Moon, a 2 next to the word Hat, and a 3 next to the word Turkey in the column (Recall A1).

-Directions (Recall A2-A5): *“Now I’m going to read the same list again, and once again when I stop I want you to tell me as many words as you can remember, including words you said the first time. It doesn’t matter in what order you say them. Just say as many as you can remember, whether or not you said them before.”*

-Administer Trial 2 and record responses in order in column (Recall A2) of the administration sheet.

-Repeat above directions and administration for trials 3, 4, and 5 and record responses in the corresponding columns labeled (Recall A3), (Recall A4), and (Recall A5).

-Directions (Recall B1): *“Now I’m going to read a second list of words. This time, again, you are to say back as many words of this second list as you can remember. Again, the order in which you say the words does not matter. Just try to remember as many as you can.”*

-Administer List B and record responses in order in column (Recall B1) of the administration sheet.

-Directions (Recall A6): *“Now I want you to tell me as many words as you can from the first list that I read to you.”*

-Record responses in order in column (Recall A6) of the administration sheet.

Delay… (After completion of the remaining Neuropsych subtests (approximately 20 minutes), the Rey-Auditory Verbal Learning Test **DELAY** should be administered.

-Directions (Delay): *“A while ago I read a list of words to you several times, and you had to repeat back the words. Tell me the words from that list.”*

-Record responses in order in column (Recall A7) of the **DELAY** administration sheet.

-Incorrect words that the participant says should be written down on the administration sheets.

-All trials should be administered as there is no discontinue rule for this subtest.

Scoring:

-The number of correct words said during recall for each trial should be tallied and recorded in the proper column under “TOTAL” on the administration sheets.

-The total number of correct words on each trial should be transferred to the Green Sheet (Neuropsych Scoring – V6) under item 4 (Rey Auditory Learning).

-Incorrect words that are said should be disregarded in scoring.

**Digit Vigilance**

Administration: (Directions are located on every set of administration sheets)

-Directions: *“’On this test, I would like for you to cross out every six that you find like this* [demonstrate first 2 sixes]*. Go across each row as quickly as you can. You may alternate going from left to right and from right to left like this* [demonstrate with finger]*, or you may go in the same direction, whichever you prefer. I want you to cross out every six you come to in the same area, and then stop.’* [point to sample area]*.”*

-If participant seems to be unclear or if “response style is inefficient”, instructions should be reviewed by examiner.

-Directions: *“’I would like you to do the same thing now on the rest of this page and tell me when you are done with that one. Then I will hand you a second page for you to do the same thing. Cross out every six as quickly as you can. Remember to go quickly, but also try to be accurate and not miss sixes. Ready? Begin!’”*

-“[If during first 5 test rows subject makes fewer than 21 correct responses, encourage accuracy] – *‘Make sure you don’t go so fast that you miss sixes. Try to be accurate!’* [If subject takes more than about 40 seconds to complete first 5 test rows, encourage rapidity] – *‘Try to go as quickly as you can. It’s all right if you miss a few.’*”

-Stopwatch is started when examiner says “Go”, and stopped when participant vocalizes that he or she has completed each page.

-Record times to the nearest hundredth of a second.

-Participant can go back to cross out missed sixes and review the page. If participant does do this, examiner should say, “Just make sure you tell me when you finish.”

-Discontinue if participant takes 400 or more seconds (6 minutes, 40 seconds) to complete Page 1.

Scoring:

-There are 103 sixes on each page.

-The number of omissions and commissions should be tallied for each page using the clear plastic (overhead) scoring key.

-Record the times and the number of omissions and commissions on both the Digit Vigilance Test administration page and also on the Green Sheet (Neuropsych Scoring – V6) under Item 5 (Digit Vigilance).

-If subtest is discontinued, write the maximum time limit (400 seconds or 6 min 40 sec on Page 1) on the Green Sheet and record the number of errors (omissions/commissions) up to the point of discontinuation.

**Trail Making Test**

Administration: (Directions are located on every set of administration sheets)

-Directions:

Trails A (Sample): *“I want you to draw a line connecting the numbers in order from 1 to 2 to 3 to 4* [trace path from number to number] *and so on, until you reach the end* [point]*. Do it as quickly as you can. Ready? Go.”*

-If participant does not understand or needs more practice, examiner should record the time and errors for each practice trial on the administration sheet.

Trails A (Test): *“Now I want you to do the same thing. This time there are more numbers* [hand Trails A sheet to subject] *so you would connect 1 to 2 to 3 to 4* [very quickly trace path] *and so on all the way to the end at 25* [point]*. Neatness does not count. Remember, work as quickly as you can, and be sure to do the numbers in order. Ready? Go.”*

Trails B (Sample): *“This one is a little different. This one has both numbers and letters and I want you to alternate – number, letter, number, letter. So you would start at 1 and draw a line from 1 to A,* [trace path]*, A to 2, 2 to B, B to 3, 3 to C, and so on, until you reach the end* [point]*. Remember, go number, letter, number, letter as quickly as you can.”*

-If participant does not understand or needs more practice, examiner should record the time and errors for each practice trial on the administration sheet.

Trails B (Test): *“Now I want you to do the same thing. This time there are more numbers and more letters* [hand Trails B sheet to subject]*. Start here at 1 and draw a line from 1 to A, A to 2, 2 to B, B to 3, 3 to C* [trace path]*, and so on until you reach the end at 13* [point to 13]*. Remember to do the numbers and letters in order by alternating number, letter, number, letter. Do this as quickly as you can. Ready? Go.”*

-Stopwatch is started when examiner says “Go.”

-Times are recorded for all trials (even samples) to the nearest hundredth of a second on the Trails administration sheet.

-All tests should be discontinued after 300 seconds (5 minutes).

-If participant makes a mistake, examiner should say “No” and instruct participant to return to the point at which the error was committed. Participant should correct the error and move on.

Scoring:

-Times and the number of errors committed during each trial should be recorded on the Trail Making Test administration sheet and also on the Green Sheet (Neuropsych Scoring – V6) under Item 6 (Trail Making).

-If a trial is discontinued, write the maximum time limit (300 seconds) on the administration sheet and the Green Sheet.

**Stroop Test – AHAB**

"Stroop - Golden Version

This version of the Stroop consists of 3 pages, each with 100 words in 5 columns of 20 items. On page 1(Word card), the words red, green, and blue are presented in black ink. On page 2 (Color card), blocks of X's are printed in red, green, or blue ink. Page 3 (Color-Word card) contains color words printed in non-congruent colors (i.e. the word blue printed in red ink, etc.). The score is the number of correctly identified items per page within 45 seconds. Errors are not counted, but the examiner should point out errors to subject during administration."

Administration: (Directions are located on every set of administration sheets)

-Directions (Words): *“This is a test of how fast you can read the words on this page. After I say ‘Begin’ you are to read down the columns starting with the first one* (point to left column) *until you complete it* (run hand down column) *and then continue without stopping down the remaining columns in order* (run hand down remaining columns)*. If you finish all the columns before I say ‘Stop’ then return to the first column and begin again. Remember, do not stop reading until I say ‘Stop,’ and read out loud as quickly as you can. If you make a mistake, I will say ‘No’ to you. Correct your error and continue without stopping. Are there any questions?* (Instructions may be paraphrased or repeated until subject understands)*. Ready? Go!”*

-Directions (Colors): *“This is a test of fast you can name the colors on this page. You will complete this page just as you did the previous page, starting with the first column. Remember to name the colors out loud as quickly as you can.”*

-Directions (Color/Words): *“This page is like the page you just finished. I want you to name the color of the ink the words are printed in, ignoring the word that is printed in each item. For example,* (point to first item of first column) *this is the first item; what would you say?*

-(If incorrect, examiner should say): *No, that is the word that is spelled there. I want you to say the color of the ink the word is printed in. Try this one* (point to next item)

-(If correct, say) *Good, you will do this page just like the others, starting with the first column* (point) *and then going on to as many columns as you can. Remember, if you make a mistake you must correct it and go on. Are there any questions? Ready, Go!”*

-Stop watch is started when participant says first word.

-Stop watch is stopped at 45 seconds.

-Examiner should follow along with participant on Task 1 (Words), Task 2 (Colors), and Task 3 (Color/Word) administration sheets.

-Examiner should make a mark on each administration sheet at the point in which the task was discontinued (when examiner says ‘Stop’ after 45 seconds of testing). This will tell the examiner the number of items that were completed during each task. This number should be recorded on the administration sheet.

Scoring:

-As noted above, the number of items completed for each task should be recorded on the data administration sheet and also on the Green Sheet (Neuropsych – V6 Scoring) under Item 7 (Stroop) for (Word 45” [Item 7A]), (Color 45” [Item 7D]), and (Color/Word 45” [Item 7G]) respectively.

-If participant is ≥ 45 years of age, the raw scores obtained should be age-corrected. For detailed instructions of this procedure, refer to loose sheet titled Appendix B (Determining T Scores) for Older Adults (45-64) (p. 31)

-In summary, if participant is ≥ 45,

- 8 points should be added to the Words raw score.
- 4 points should be added to the Colors raw score.
- 5 points should be added to the Color/Words raw score.

-T-Scores should be obtained from “Table 1-B T Scores for Stroop Data” on page 31 (Appendix B).

-If participant is younger than 45, the raw scores should be used to determine T Scores.

-If participant is 45 or older, the age-corrected score should be used to determine T Scores.

-If raw score does not exactly match a T-score, score the T-score it’s closer to. Example: Raw score on color trial is 78. Score with the T-score associated with raw score 77 (i.e. 48). If raw score falls in the middle, go with the middle T-score (example raw score on word trial is 98. Available T-scores are 44 (raw=96) or 46 (raw=100). Score as T-score=45.)

- If the T-score falls below 20, then score as 20. If the T-score falls above 99, then score as 99.

- If the participant is color-blind, and the sheets are not administered, record –9999 on the data entry sheet and make a note in the comments section.

-All raw scores, age-corrected scores, and T-Scores should be recorded on Stroop Test – AHAB administration sheet and on the Green Sheet (Neuropsych Scoring – V6) under Item 7 (Stroop) in their proper places.

**General Neuropsych Guidelines:**

**Record all scores on the Neuropsych Scoring Sheet (the Green Sheet) (see attached). Note any behavioral observations on the scoring summary sheet for data entry in the Comments section. For example, subject was tired, color-blind, or uncooperative. Also make sure to note on data entry sheet if the participant is a non-native English speaker. Record participant’s native language and how long he or she has been speaking English on a daily basis.**

6. **Wechsler Abbreviated Scale of Intelligence** (WASI):

*Next we’ll do a couple of activities that involve defining words and solving problems. Most people don’t answer every item correctly or finish every item, but please give your best effort on all the items. Do you have any questions?*

## WASI SUBTESTS

1. **Vocabulary**

- See WASI Scoring Manual

1. **Block Design**

- Stopwatch is started when tester says go

- Stopwatch is stopped when participant says they are done

- Scramble the blocks between designs

- Make sure to check for rotation errors

- Keep stimulus book approx. 7 inches from and parallel to the edge of the testing table

- See WASI Scoring Manual for additional administration/scoring information

1. **Similarities**

- See WASI Scoring Manual

1. **Matrix Reasoning**

- See WASI Scoring Manual

## *- Allow subject to change answer up to the start of the next item*

Record scores on the Neuropsych Scoring Sheet.

Note any behavioral observations on the scoring summary sheet for data entry in the Comments section. For example, subject was tired, color-blind or uncooperative.

Also make sure to note if the participant is a non-native English speaker. Record their native language, and how long they have been speaking English on a daily basis.

7. **Sensus** (CESD, STAI, CMHI, STAXI, BPAQ, HS, MCSD, LOTR, SRA, MAAS

Questionnaires are completed on the three (3) computers located in room 507. Start menu, Programs, Sensus, Run Sensus Q&A 2.0. Choose v1.qar from main menu; click ‘start’, enter subject ID (AHAB ID). Record ID number, date and type of questionnaire on appropriate computer log sheet.

If subject is unable to finish the Sensus questionnaires, he or she can stop at the instruction page of any of the individual questionnaires. Each instruction page has a button labeled quit. Click on the Quit button and make a note in the protocol folder and in the Sensus Mid folder hanging on the wall by 507A.

**Fish Oil Chart Documentation**

| **Appointment** | **Task** |
| --- | --- |
|  | |
| **V6b** | 1. Verify eligibility 2. Eligibility Verification sheet (started at V1 of AHAB) 3. Tracking folder appointment sheet 4. New medications and illnesses 5. Fill out paperwork 6. Review Consent and sign 7. Side effects 8. Review compliance/dosing information 9. Schedule appointments 10. Dispense fish oil (2 extra packs) 11. Document 12. # packets and pills dispensed 13. Lot number 14. Expiration date |
|  | |
| **V7** | 1. Schedule appointments 2. Collect and count F/O returned 3. Vital signs 4. Side effects 5. Dispense new packets 6. Document 7. # packets and pills returned 8. # packets and pills dispensed (include return of partial pack) 9. Lot number (verify it matches first lot number at V6b) 10. Expiration date 11. Calculate and document compliance |
|  | |
| **V8** | 1. Screen for contraindications of MRI scan (metal, pregnancy) 2. N-back 3. Flanker |
|  | |
| **V9** | 1. ED training 2. Sensus (NEO, STAXI, CMHI, BIS, BPAQ, PSS, DAS, BDI) 3. Changes questionnaire |
|  | |
| **V10** | 1. Vital signs 2. New medications and illnesses 3. Side effects 4. Blood draw 5. Draw green tube if had at V1 (check tracking folder) 6. Height and weight 7. Collect and count F/O returned 8. Document 9. # packets and pills returned 10. Calculate compliance 11. Chart to front desk for Dr. Muldoon with Fish Oil hand out |

**Fish Oil Supplementation**

**Visit 6b (½ hour)**

RESTRICTIONS:

None.

PROTOCOL:

- Informed Consent
- Med History and Medication Recheck
- Side Effect Questionnaire
- Randomization and distribution of fish oil capsules
- Schedule 2-week follow-up phone call

1. **Informed Consent**
2. Review study, visit procedures, and risks/benefits
3. Allow subject to ask questions and answer completely and honestly so that subject understands study and the scope of their involvement.
4. When subject agrees upon participation, have them initial each page of the consent form and sign and date the final page; RA must also sign and date.
5. Document *Informed Consent Process;* file in tracking folder (left side) and on Protocol Adherence

Give copy of signed consent form to participant

Original filed in OEH lab (504)

1. **Medical History and Medication recheck**

Completed by nurse to determine if new medical condition or medication effects eligibility

1. **Side Effect questionnaire**

Subject should self-report, follow-up questions as necessary.

1. **Randomization**

Contact Janet Lower (4-7728) with schedule ID, sex, race, age

Logon: R-track (<https://haller.nursing.pitt.edu/crcd/>)

Select ‘Randomize’, P01 Study 3 (Muldoon)

Enter Subject ID: (scheduling ID)

Chose: Race, Gender, Age

Select Randomize

Document group randomization in Excel spreadsheet (idlist)

Deliver 9 packs to study nurse

Review procedures for taking fish oil and adherence.

1. **Schedule 2-week follow-up phone call**

Open *Copy of Fish Oil Timeline.xlsx* found on T:\Shared Docs

Enter scheduling ID

Enter V6b date which will calculate ***due*** dates

Enter 2-week follow-up on *STUDY CALENDAR*

**Schedule V7**

Open *Copy of Fish Oil Timeline.xlsx* found on T:\Shared Docs

Find scheduling ID

Enter TC scheduled date which will calculate V7 due date

Enter V7 date on *STUDY CALENDAR*

**2-week Follow-up Phone Call**

PROTOCOL:

- Adherence/Side Effect (Compliance/Questionnaire)
- Determine if additional follow-up phone call is needed
- Schedule V7

| **2-week Follow-Up phone call** | |  | | | Initials | Comments |
| --- | --- | --- | --- | --- | --- | --- |
|  | Compliance |  Y |  N |  Other |  |  |
|  | Side Effect Questionnaire |  Y |  N |  Other |  |  |
|  | Add’l follow-up scheduled |  Y |  N |  N/A |  |  |
|  | V7 scheduled/confirmed |  Y |  N |  Other |  |  |

1. **Adherence/Side Effect Script**

This is ________________from the AHAB fish oil supplement study. I’d like to ask you some questions; do you have a few minutes?

1. How is the fish oil study going for you?
2. I’m going read a list of potential side effects; if you have the side effect, please rate the severity as none, mild, moderate, or severe (refer to side effects questionnaire).
3. Most people have some difficulty remembering to take pills daily, have you missed taking any doses in the past 2 weeks?

_____No (skip to #4) _____Yes

If yes:

- 1. In the past 2 weeks, how many capsules did you miss taking? (daily dose is 2 capsules)

_____doses ___#days since Rx began x 2=___#pills expected

___#expected pills-___pills missed / #expected x100%= ___ % compliance

93-100% No intervention

81-92% Intervention (refer to fish oil guidelines and adherence strategies)

≤ 80% Provide intervention and schedule additional telephone call in 2 weeks

- 1. What are the reasons you missed taking the doses?

__________________________________________________________________________________

4. Do you have a special routine or reminder system that you use to remember to take your doses daily?

_________________________________________________________________________________________

*RN*

*Schedule or Remind participant of next appointment or telephone call*

*Date: __________________Time:_______________________*

1. **Schedule V7**

Open *Copy of Fish Oil Timeline.xlsx* found on T:\Shared Docs

Find scheduling ID

Enter TC scheduled date which will calculate V7 due date

Enter V7 date on *STUDY CALENDAR*

**Visit V7 (½ hour)**

RESTRICTIONS:

Abstain from caffeine, tobacco products and exercise for 2 hours prior to appointment

Refrain from drinking alcohol the day of the appointment

PROTOCOL:

- Blood Pressure
- Compliance
- Side Effect Questionnaire
- Distribution of fish oil capsules
- Schedule 12-week follow-up phone call
- Schedule V8 & V9

1. **V7 Restrictions**
2. **Blood pressure cuff measurement**
   1. Blood pressure measurement procedure is explained to the participant
   2. Using the left arm, measure mid-arm circumference to determine proper cuff size*
   3. Record on blood pressure measurement form.
   4. Participant is asked to remain seated, legs uncrossed, for a period of 5 minutes (set timer) and during blood pressure measurement. Participant may fill out questionnaires or answer minimum benign questions.
   5. Brachial artery is located and the cuff is placed snugly on the left arm: bladder is centered over the brachial artery, 1 -1/1/2 inches above the crease.
   6. Arm is positioned at heart level

*Arm circumference measurement: Ask participant flex left arm at a 90 angle and measure the length between the acromiun (shoulder) and olecranon process (elbow) to determine midpoint of arm length. Have the participant relax arm and dangle at side. Place tape measure around determined midpoint of arm to obtain arm circumference. Select cuff size according to chart below.

| **Circumference** | **Cuff size** |
| --- | --- |
| < 23 cm | Small |
| 23 – 30 cm | Regular |
| > 30 – 37.5 cm | Large |
| > 37.5 cm | Thigh |

1. **Blood Pressure**
   1. Wait 5 minutes, determine maximum inflation level (MIL):
      1. Palpate radial pulse and inflate the cuff to approximately 70 mm Hg. Continue to inflate cuff at increments of 10 mm Hg until the pulse disappears to touch. Deflate the cuff.
      2. Remember the level on the manometer when the pulse disappears and add 30 mm Hg at the next BP measurement.
   2. Brachial artery pulse is palpated and stethoscope bell (or diagphram) is placed over the pulse.
   3. Cuff is inflated to MIL.
   4. Cuff is deflated at about 2 mm Hg/sec while listening for first and last K sounds.
   5. Cuff is rapidly deflated after last sound.
   6. Blood pressure reading is recorded.
   7. Take 30 second radial pulse (record as beats per minute)
   8. Steps b – f are repeated for second blood pressure
   9. The cuff is removed from the participant’s arm
2. **Side Effect questionnaire**

Subject should self-report, follow-up questions as necessary.

1. **Distribution of additional pills**

Contact Janet Lower (4-7728) with schedule ID and number of additional pill packs needed to finish study

Look up schedule ID in Excel spreadsheet (idlist) to determine randomization group

Deliver additional packs to study nurse

1. **Schedule 12-week follow-up phone call**

Open *Copy of Fish Oil Timeline.xlsx* found on T:\Shared Docs

Find scheduling ID

Enter V7 date which will calculate ***due*** date

Enter 12-week follow-up on *STUDY CALENDAR*

**Schedule V8/V9**

Open *Copy of Fish Oil Timeline.xlsx* found on T:\Shared Docs

Find scheduling ID

Enter V7 which will calculate V8/V9 due dates

Enter V8 &V9 dates on *STUDY CALENDAR*

**12-week Follow-up Phone Call**

PROTOCOL:

- Adherence/Side Effect (Compliance/Questionnaire)
- Determine if additional follow-up phone call is needed
- Schedule V7

**Adherence/Side Effect Script**

This is ________________from the AHAB fish oil supplement study. I’d like to ask you some questions; do you have a few minutes?

1. How is the fish oil study going for you?
2. I’m going read a list of potential side effects; if you have the side effect, please rate the severity as none, mild, moderate, or severe (refer to side effects questionnaire).
3. Most people have some difficulty remembering to take pills daily, have you missed taking any doses in the past 2 weeks?

_____No (skip to #4) _____Yes

If yes:

- 1. In the past 2 weeks, how many capsules did you miss taking? (daily dose is 2 capsules)

_____doses ___#days since Rx began x 2=___#pills expected

___#expected pills-___pills missed / #expected x100%= ___ % compliance

93-100% No intervention

81-92% Intervention (refer to fish oil guidelines and adherence strategies)

≤ 80% Provide intervention and schedule additional telephone call in 2 weeks

- 1. What are the reasons you missed taking the doses?

__________________________________________________________________________________

4. Do you have a special routine or reminder system that you use to remember to take your doses daily?

_________________________________________________________________________________________

*RN*

*Schedule or Remind participant of next appointment or telephone call*

*Date: __________________Time:_______________________*

**Visit V8 (2 hours)**

RESTRICTIONS:

Abstain from caffeine, tobacco products and exercise for 3 hours prior to appointment.

Refrain from drinking alcohol and taking non-essential medications for 12 hours prior to appointment.

Participants will be asked about any non-removable medical devices or implants, and about any other sources of metal in or on their bodies. In cases of suspected prior exposures or implants that would pose a risk for magnetic resonance imaging, such participants will receive a plain film x-ray exam prior to the MRI study. This x-ray exam will determine the amount and location of any metal within the participant’s body, and thus, whether the participant can be safely scanned in the MRI.

PROTOCOL:

- Screening for contraindications of MRI scans
  - Metallic objects
  - Pregnancy
- N-Back and Flanker tasks in MRI scanner

**Visit V9 (3 hours)**

RESTRICTIONS:

None.

PROTOCOL:

- ED training
- Changes questionnaire
- Sensus
  - NEO
  - STAXI
  - CHMIH
  - BIS
  - BPAQ
  - PSS *(added to protocol 10/3/2008)*
  - DAS *(added to protocol 10/3/2008)*
- BDI

**Visit V10 (4 hours)**

RESTRICTIONS:

Nothing to eat or drink, except water, after 9 p.m. night before visit. Includes no caffeine 2 hours prior to visit and no alcohol day of visit

No tobacco 2 hours prior to visit

No exercise 2 hours prior to visit

PROTOCOL:

- Blood Pressure
- Med History and Medication Recheck
- Compliance
- Side Effect Questionnaire
- ED return
- Blood draw
- Lipids, CPR/IL-6, Fatty Acids, Immune Functions (*(added to protocol10/23/09)*
- HRV
- Endothelial Function *(added to protocol4/2009; removed from protocol 6/16/10 – no subjects completed )*
- DDT
- SI-TABP
- Neuropsych
- DPX
- WASI
- FFQ
- Payment

**V9 Restrictions**

1. **Medical History and Medication recheck**

Completed by nurse to determine if new medical condition or medication effects eligibility

1. **Blood pressure cuff measurement**
   1. Blood pressure measurement procedure is explained to the participant
   2. Using the left arm, measure mid-arm circumference to determine proper cuff size*
   3. Record on blood pressure measurement form.
   4. Participant is asked to remain seated, legs uncrossed, for a period of 5 minutes (set timer) and during blood pressure measurement. Participant may fill out questionnaires or answer minimum benign questions.
   5. Brachial artery is located and the cuff is placed snugly on the left arm: bladder is centered over the brachial artery, 1 -1/1/2 inches above the crease.
   6. Arm is positioned at heart level

*Arm circumference measurement: Ask participant flex left arm at a 90 angle and measure the length between the acromiun (shoulder) and olecranon process (elbow) to determine midpoint of arm length. Have the participant relax arm and dangle at side. Place tape measure around determined midpoint of arm to obtain arm circumference. Select cuff size according to chart below.

| **Circumference** | **Cuff size** |
| --- | --- |
| < 23 cm | Small |
| 23 – 30 cm | Regular |
| > 30 – 37.5 cm | Large |
| > 37.5 cm | Thigh |

1. **Blood Pressure**
   1. Wait 5 minutes, determine maximum inflation level (MIL):
      1. Palpate radial pulse and inflate the cuff to approximately 70 mm Hg. Continue to inflate cuff at increments of 10 mm Hg until the pulse disappears to touch. Deflate the cuff.
      2. Remember the level on the manometer when the pulse disappears and add 30 mm Hg at the next BP measurement.
   2. Brachial artery pulse is palpated and stethoscope bell (or diagphram) is placed over the pulse.
   3. Cuff is inflated to MIL.
   4. Cuff is deflated at about 2 mm Hg/sec while listening for first and last K sounds.
   5. Cuff is rapidly deflated after last sound.
   6. Blood pressure reading is recorded.
   7. Take 30 second radial pulse (record as beats per minute)
   8. Steps b – f are repeated for second blood pressure
   9. The cuff is removed from the participant’s arm
2. **Blood Draw**

Participants will be asked to abide by the following restrictions:

Must fast from 9pm the evening before this appt.; nothing to eat or drink except water

(Asked to abstain from using tobacco and caffeine for two hours prior to visit and refrain from exercise for two hour prior to this visit)

The following tubes should be drawn in the order below.

| **Tube & volume** | **Priority** | **Processing** | **Use** |
| --- | --- | --- | --- |
| 3.15 ml blue top  (22-040-044) | 1 | Remove 1 ml whole blood  Place in lab refrigerator  Deliver to Genetics lab | 1 ml whole blood, stored at -70^o^C  RBC sample for fatty acid analysis.  Store in original vacutainer tube |
| Two 4.5 ml blue tops  (02-688-26) | 2 | Room temperature – 20 min  *Centrifuge 10 min; aliquot plasma*  Deliver remainder to Genetics lab. | Plasma: .5 ml for IL6 by Marsland lab,  .5 ml for CRP (University of Vermont)  3 citrate storage samples @ ≥.9 ml. |
| 10 ml red/grey top  (02-683-98) | 3 | Room temperature, allow 20 min to clot  *Centrifuge 10 min*  Deliver to Genetics lab | 1.5 ml for lipids, insulin and glucose  plus  3 serum samples @ ≥.9 ml each for storage |
| 10 ml purple top  (02-657-32) | 4 | Room temperature  Deliver to Genetics lab | 3 EDTA plasma samples @ ≥ .9 ml each for storage |
| 6 ml green top  (02-687-97) | 5 | Room temperature  Deliver to Marsland lab | Whole blood stimulated cytokine production |

Fill all tubes completely.

Record time of blood draw on Blood Draw Eligibility form.

Document any difficulties with blood sampling (this would include tubes that are incompletely filled or are not obtained) and which tubes are successfully drawn on the AHAB-II blood tracking form.

1. **ED Compliance**

Determine compliance % for electronic diary monitoring

Step 1: Getting to the feedback screen

1. Normally, the ED should be either on the Palm main screen (shows icons for all the Palm functions) or on the intro screen for the monitoring program (has the buttons to quit, report a cigarette, put ED to sleep, quiet mode, etc.).

***YOUR GOAL FOR STEP 1 IS TO REACH THE “PLEASE CALL BMRG” SCREEN.
WHEN YOU SEE THIS, YOU’RE READY FOR STEP 2.***

- 1. **If ED is on Palm main screen** tap the PPG_ED_proj3 icon (a red circle with a white stylus, generally in the lower right corner of the screen). You do not need to enter a subject ID or schedule on the next screen, just tap Next to proceed. You may have to click through a Beginning of Day interview. If this occurs, just tap Next to move through the screens, selecting any multiple choice answer when required (this interview will be cut off from the participant’s data). The ED screen will power down after the interview is complete, but if you hit the power button (with green dash, on lower left of ED). See ‘c.’
  2. **If ED is anywhere in an interview,** complete the interview with dummy answers to return to the monitoring main menu (intro screen). See ‘c.’
  3. **If at monitoring main menu (intro screen),** tap the “Help” button. On the next screen tap “Contact BMRG.”
  4. **If the ED won’t turn on:** As a last resort, if there is no response from tapping the screen or pressing the power button, turn the ED over and use the stylus to press the small Reset button on the back of the device. This will not erase the participant’s data. Once the Palm activates, you will have to set the date and time zone then proceed to the main screen by tapping the icon that looks like a little house [below the display screen]. See ‘a.’

Step 2: The “hidden” button, calculating the access code

1. On the “call BMRG” screen, tap the upper left corner (the **hidden button**). This should take you to a screen where you can select Shakedown Report or Feedback Report. Select Feedback and tap Next. A new screen should appear asking you to enter a code.
2. The **access code** is derived from the “day” number in the current date and the hour in military time. Essentially, the code is derived from adding the day to the military hour. The trick to the code is that even numbers for day or hour values are divided by two before adding. See examples below.

**Example 1 (both odd numbers): 3:11pm on October 21^st^** 15 + 21 = 36
The code would be 36.

**Example 2 (both even numbers): 10:26am on December 12^th^**

(10/2) + (12/2) = 5 + 6 = 11

The code would be 11.

**Example 3 (odd date, even hour): 2:04pm on July 7^th^**

(14/2) + 7 = 7 + 7 = 14

The code would be 14.

1. To input the code, tap the ovals on the screen containing zeros. Tapping will increment the tapped digit by one.

Step 3: Reading the output, determining bonus payment

1. The feedback output will tell you more information than you actually need (stuff about the modes for each day of monitoring, as well as the current mode, along with the number of missed interviews). What you really is “xx% complete,” which should appear right in the center of the screen below “Interview Summary.”

For unexpected errors: Call Teresa, Melissa or Natalie at BMRG

Teresa Steigerwalt, BMRG data manager, (412) 624-2097

Melissa Delaney, AHAB-II research assistant, (412) 383-5029

Natalie Sevilla, AHAB-II research assistant, (412) 383-6945

BMRG AHAB-II cell phone (if no one can be reached at an office phone), (412) 576-9069

1. **Side Effect questionnaire**

Subject should self-report, follow-up questions as necessary.

1. **HRV (RSA)**

Setup computers for data collection

1. On Gateway Computer, login

-username = AHAB

-password = testing

1. Double click “ACQ 8.0” Icon to open MindWare
2. Click “Start”
3. Click “Acquire data”
4. Select “HRV Data” Folder
5. At prompt “Please enter a filename”, enter “AHABIDFOur”
6. Hit “Enter” or click “OK”
7. Switch to Mr. Data Computer using KVM switch
8. Mr. Data monitor should be set on “Reactivity” (default), hit “Enter”
   1. At C:\AHAB prompt type “ahab&”, then hit “Enter”
   2. Enter subject’s “AHAB ID”, then hit “Enter”
   3. Select “1” (RSA), then hit “Enter”
   4. Hit “Enter” again
   5. Check to make sure settings at the bottom of the screen are correct (blue print at bottom of screen)
      - You should NOT need to change anything
      - Rate = 1 Khz
      - a(5-digit ID)FOur
      - #Channel = 2
      - Test time = 300 (seconds)
      - Rest time = 0
      - Cycle time doesn’t matter, it can be anything
9. Switch to Gateway Computer using KVM switch

Computers are now ready for data collection.

*“Do you need to use the restroom before we get started in the lab?”*

*“Do you have a cell phone or pager on you today? We’re going to ask that you turn it off while we’re in the lab today because it may interfere with some of the monitoring devices that we use.” (Pt. can leave phone with RA while in Chamber 2 if they refuse to turn off device).*

ECG Overview & Preparation

Lead pt. into chamber

*-Ask medication questions & segue into ECG explanation.*

*“We will be performing an ECG or electrocardiogram. This will record electrical signals from your heart. The ECG is a simple non-invasive procedure. I will simply place three sensors on the surface of your skin, one on each wrist and one on your left ankle. During this procedure you will sit still with this respiration belt around your waist and your arms & legs will be still with your palms facing up. I will need to rub your wrists and ankle with some alcohol, and then the sensors will go on just like Band-Aids.”*

*“If you will, please stand for me so that I can put this respiration belt around your waist. It will measure your breathing.”*

- Place respiration belt around abdomen so that it fits snugly while subject is seated
- Confirm from subject that belt is snug but not too tight when seated
- Squeeze bulb 1or 2 times (may need to be adjusted if respiration signal is small)

*We will begin with a five-minute resting period. During this time, I will need you to sit as still and as quiet as possible. After the first five minutes, there will be another five-minute resting period during which I will again ask you to be as still and as quiet as possible. However, for the second five-minute period, I will ask you to breathe according to a regular pattern. I’ll explain this to you in more detail before we begin that portion of the session.”*

*“Do you have any questions so far?”*

- Put gloves on
- Rub wrists & left ankle with alcohol and gauze and attach wrist & ankle sensors for ECG

Sensors should be attached as follows:

*RA* = *Right Wrist*

*LA* = *Left Wrist*

*LL* = *Left Ankle (ISO Ground)*

RSA Breathing Tasks

*“We are now ready to begin the first five-minute rest period. Remember to sit as still and as quietly as you can. Go ahead and move or adjust to get comfortable, but be careful not to pull any of the wires or belt loose. Let me know when you are comfortable so that I can check the signals on the computer. I will be right outside the door during the measurements and there is a microphone in this room in case you need anything.”*

- Once subject indicates s/he is comfortable, check respiration signal on MindWare (Gateway) by clicking “Start”—the graph should look like a sine wave (note if wave is shallow, it may be difficult to score later so you’ll want to be sure that you can clearly see breathing motions)
- Adjust belt or bioamplifier as needed to obtain visible respiration signal
- Click “Stop” on MindWare (Gateway)
- Click “Exit”
- Click “Acquire Data”
- Select “HRV Data” Folder
- Select file with “AHABIDFOur”
- Computer will ask if you want to replace the file…Select “Replace”
- Switch to Mr. Data Computer

*“The signals look good so we can start. Again, just sit still and breathe as you normally would. Please don’t talk and just do your best to stay awake. I’ll let you know when we’re finished. Ready?…Begin”*

- On Mr. Data keyboard hit F1
- Switch to Gateway
- Click “Start” on MindWare (Gateway)
- Mr. Data will stop collection after 5 minutes
- Click “Stop” on MindWare (Gateway)

*“Okay, we’re finished with the first five-minute rest period. Just give me one minute to setup the computer and I’ll be in to give you instructions for the second rest period.”*

- Click “Exit” on MindWare
- Click “Acquire Data”
- Select “HRV Data” Folder
- Type “AHABIDFOpr”
- Switch to Mr. Data computer
- Walk around to where subject is to give instruction for paced respiration

*“We are now ready to move onto the second rest period. This time I would like you to breathe according to a regular pattern. To help you do that, the computer will generate two different sets of tones over and over again. The first set of tones is higher in pitch and during this set I would like you to inhale as smoothly and evenly as you can. Then, during the lower pitch set, I would like you to exhale as smoothly and evenly as you can. You should be aware that the sets of tones are not equal in length, the inhale set has four beeps and the exhale set has five. Remember in for four, out for five. It is not necessary that you take deep breaths, just so you stay on pace. Again, I ask that you remain as still as possible. I will give you a few moments to practice and get used to the tones. Then, once I see that you are on pace, I will reset the computer and we will start the task. Any questions?”*

- If subject has no questions, return to computers and hit “Enter” on Mr. Data keyboard
- Switch to Gateway computer
- Click “Start” on MindWare
- Check to see if breaths are even (sine wave should be virtually symmetrical)
- If waves are lopsided, have subject adjust accordingly
- Once subject is able to produce 3 cycles of symmetrical waves you can begin the task
- Click “Stop” on MindWare
- Click “Exit”
- Click “Acquire Data”
- Select “HRV Data” Folder
- Select File with “AHABIDFOpr”
- Replace file?...Select “Replace”
- Switch to Mr. Data
- Hit “Enter” on Mr. Data

*“It looks as though you have the hang of it so we can get started. Are you comfortable? (allow subject to adjust if necessary). Okay, once I say begin, the computer will delay for a second, then it will start with an inhale set, followed by an exhale set at the same rate that you just practiced. This task will last 5 minutes. Again, please don’t move or talk and do your best to stay awake. Try to stay on pace and breathe as smooth and as evenly as possible. Ready?…Begin”*

- Hit “F1” on Mr. Data
- Switch to Gateway computer
- Click “Start” on MindWare
- Monitor MindWare closely to be sure subject stays on pace

1. If subject cannot achieve pace after 10 seconds, verbalize when to inhale and exhale by saying “in” and “out” for next three sets
2. If subject remains on pace for at least 90 consecutive seconds task is complete
3. If subject cannot stay on pace after first two minutes start task again (refer to trouble shooting guide for instructions on how to reset the task)

- Mr. Data will stop collection after five minutes
- Hit “2” (exit), then “Enter” on Mr. Data
- Click “Stop” on MindWare

*“Okay, we are finished with the ECG and we can remove the equipment now.”*

- Remove ECG leads and hang on wall
- Allow participant to remove adhesive sensors from wrists
- Have subject stand up and loosen valve on respiration belt, then remove

*Note: If you are running the last ECG of the day, please change the battery.*

1. **DDT** (Time discounting task - computer administered)

CLICK ON *DDT2* SHORTCUT ON SCREEN

SELECT ID FROM TOP TOOLBAR THEN ENTER SUBJECT ‘ID*FO’*

SELECT START TO BEGIN

*During this task, you will be presented with a series of choices. In each case, you will be asked to choose between a variable amount of money available NOW, and a fixed amount of money available after a delay. For each choice, the dollar amounts and delays will first be presented on the screen in white. During this time, no response will be counted. After 2 seconds, the choices will appear in black, and you will then be able to make your selection. Please consider each choice carefully, and answer the questions as if it were real money. Some questions may be repeated. Please press any key when you are ready to begin.*

1. **SI-TABP**

This is a structured interview, meaning that every participant in the AHAB study will be asked the same questions and not all of the questions will apply to everyone. I am going to record this interview so that this interview can be coded at a later day.

Record for coding using digital recorder; files will be coded at a later date by all RAs.

Save using AHAB ID number

Upload to server folder (T:\Interviews-Fish Oil)

1. **Neuropsych**

**Spatial Span**

Administration: (WMS Stimulus Booklet 1)

*Forward*:

-Place Spatial Span Board on testing table.

-Directions: *“Now I want you to do exactly what I do. Touch the blocks I touch, in the same order.”*

-Touch blocks with pen/pencil at rate of one block per second. Record the order in which participant taps blocks on Spatial Span administration sheet.

-Adhere to discontinuation criterion on Spatial Span administration sheet if applicable.

-Allow participant to change answer up to the start of the next trial.

*Backward*:

-Directions: *“Now I am going to touch some more blocks. This time when I stop, I want you to touch the blocks backward, in the reverse order of mine. For example, if I touch this block* (Cube 3)*, then this one* (Cube 5)*, what would you do?”*

-If participant responds correctly, say *“That’s right”* and remind participant to touch the blocks in reverse order, then move on to the first trial of Item 1.

-If participant responds incorrectly, examiner should say *“No, I touched this one, then this one;…”* and instruct participant how to do the subtest correctly as per directions in the Stimulus Booklet 1 Spatial Span Backward section.

-Touch blocks with pen/pencil at rate of one block per second. Record the order in which participant taps blocks on Spatial Span administration sheet.

-Adhere to discontinuation criterion on Spatial Span administration sheet if applicable.

-Allow participant to change answer up to the start of the next trial.

Scoring:

One point is awarded on Spatial Span administration sheet for every trial of every item that is correct. The total points are then tallied and recorded on administration sheet under “Forward Total Score” and “Backward Total Score” and “Total Score”.

Scores should be transferred to Green Data Sheet (Neuropsych Scoring – V6) under Item 1 (Spatial Span). Age-scaled scores should be derived from Table D.1 of the WMS –III Administration and Scoring Manual (p. 139-141) and should be entered on the Green Sheet.

**Digit Span**

Administration: (WMS Stimulus Booklet 1)

*Forward*:

-Directions: *“I am going to say some numbers. Listen carefully, and when I am through, I want you to say them right after me. Just say what I say.”*

-Read digits at rate of one per second. WMS administration booklet instructs examiner to “drop your voice inflection slightly on the last digit in the sequence.”

-Record order in which participant says numbers on Digit Span administration sheet.

-Adhere to discontinuation criterion on Digit Span administration sheet if applicable.

-Allow participant to change answer up to the start of the next trial.

*Backward*:

-Directions: *“Now I am going to say some more numbers. But this time when I stop, I want you to say them backward. For example, if I say 7-1-9, what would you say?”*

-If participant responds correctly, say *“That’s right”* and move on to the first trial of Item 1.

-If participant responds incorrectly, examiner should say *“No, you would say 9-1-7…”* and instruct participant how to do the subtest correctly as per directions in the Stimulus Booklet 1 Digits Backward section.

-Read digits at rate of one per second.

-Record order in which participant says numbers on Digit Span administration sheet.

-Adhere to discontinuation criterion on Digit Span administration sheet if applicable.

-Allow participant to change answer up to the start of the next trial.

Scoring:

One point is awarded on Digit Span administration sheet for every trial of every item that is correct. The total points are then tallied and recorded on the administration sheet under “Forward Total Score” and “Backward Total Score” and “Total Score”.

Scores should be transferred to Green Data Sheet (Neuropsych Scoring – V6) under Item 2 (Digit Span). Age-scaled scores should be derived from Table D.3 of the WMS –III Administration and Scoring Manual (p. 172-177) and should be entered on the Green Sheet.

**Short-term Memory Test (Four-word Memory Test)**

Administration: (Directions are located on every set of administration sheets)

Directions: *“’I’m going to read you four words, which I would like you to try to remember. In order to make your task more difficult, however, after I read the fourth word, I’m going to read a 3-digit number, like 100. As soon as I read you that number, I want you to begin counting backwards by threes as rapidly and as accurately as you can. I want you to continue doing that until I tell you to stop. At that point you’ll tell me what the four words are.’*

*‘How good are you at counting backwards by threes? Let’s try it. Start from 100.’ [provide practice – let subject count to approximately 70]*

*‘Before we begin, I want to quickly review what you’ll be doing. First, you’ll hear 4 words – and I want you to try to remember those. Then you’ll hear a number – and I want you to count backwards from it by threes. After a while, I’ll tell you to stop, and you’ll tell me the words.’*

*‘Here are the first four words I want you to try to remember…’”*

-Read words at rate of one per second.

-Upon participant recall after examiner says “Stop,” circle correct words and notate the order in which they are said.

-Draw a line through words that are not said by participant.

-Write incorrect words in the space provided for “incorrect response” on administration sheet.

-There is no discontinue rule, so all items should be administered.

Scoring:

-Tally all correct words (circled) for each of the three times and record the number of correct words in the table on the bottom of the second administration sheet in the column labeled (#) for 5 seconds, 15 seconds, and 30 seconds respectively. Add these items to determine the total number of correct words and record this number on the administration sheet as well.

-Tally all incorrect words that are written in the “incorrect response” column for each of the three times and record these numbers in the table on the bottom of the second administration sheet in the column labeled (I) for 5 seconds, 15 seconds, and 30 seconds respectively. Then, add the (I) items to determine the total number of incorrect responses and record this number in the table.

-Disregard columns (P) and (E) of the table.

-Scores should be transferred to the Green Sheet (Neuropsych Scoring – V6) under Item 3 (Four-word Memory Test).

**Rey Auditory Verbal Learning Test**

Administration: (Directions are located on every set of administration sheets)

-Directions (Recall A1): *“I am going to read a list of words. Listen carefully, because after I stop, I want you to say back to me as many words as you can remember. It doesn’t matter in what order you repeat them. Just try to remember as many as you can.”*

-Words should be read at rate of one per second. When examiner has finished reading words, participant should be asked to say as many words as he can remember.

-Participant responses should be recorded in order on the administration sheet in the column labeled (Recall A1). A simple number in column (Recall A1) next to the word is sufficient. For example, if the participant says “Moon, Hat, Turkey” then the examiner should place a 1 next to the word Moon, a 2 next to the word Hat, and a 3 next to the word Turkey in the column (Recall A1).

-Directions (Recall A2-A5): *“Now I’m going to read the same list again, and once again when I stop I want you to tell me as many words as you can remember, including words you said the first time. It doesn’t matter in what order you say them. Just say as many as you can remember, whether or not you said them before.”*

-Administer Trial 2 and record responses in order in column (Recall A2) of the administration sheet.

-Repeat above directions and administration for trials 3, 4, and 5 and record responses in the corresponding columns labeled (Recall A3), (Recall A4), and (Recall A5).

-Directions (Recall B1): *“Now I’m going to read a second list of words. This time, again, you are to say back as many words of this second list as you can remember. Again, the order in which you say the words does not matter. Just try to remember as many as you can.”*

-Administer List B and record responses in order in column (Recall B1) of the administration sheet.

-Directions (Recall A6): *“Now I want you to tell me as many words as you can from the first list that I read to you.”*

-Record responses in order in column (Recall A6) of the administration sheet.

Delay… (After completion of the remaining Neuropsych subtests (approximately 20 minutes), the Rey-Auditory Verbal Learning Test **DELAY** should be administered.

-Directions (Delay): *“A while ago I read a list of words to you several times, and you had to repeat back the words. Tell me the words from that list.”*

-Record responses in order in column (Recall A7) of the **DELAY** administration sheet.

-Incorrect words that the participant says should be written down on the administration sheets.

-All trials should be administered as there is no discontinue rule for this subtest.

Scoring:

-The number of correct words said during recall for each trial should be tallied and recorded in the proper column under “TOTAL” on the administration sheets.

-The total number of correct words on each trial should be transferred to the Green Sheet (Neuropsych Scoring – V6) under item 4 (Rey Auditory Learning).

-Incorrect words that are said should be disregarded in scoring.

**Digit Vigilance**

Administration: (Directions are located on every set of administration sheets)

-Directions: *“’On this test, I would like for you to cross out every six that you find like this* [demonstrate first 2 sixes]*. Go across each row as quickly as you can. You may alternate going from left to right and from right to left like this* [demonstrate with finger]*, or you may go in the same direction, whichever you prefer. I want you to cross out every six you come to in the same area, and then stop.’* [point to sample area]*.”*

-If participant seems to be unclear or if “response style is inefficient”, instructions should be reviewed by examiner.

-Directions: *“’I would like you to do the same thing now on the rest of this page and tell me when you are done with that one. Then I will hand you a second page for you to do the same thing. Cross out every six as quickly as you can. Remember to go quickly, but also try to be accurate and not miss sixes. Ready? Begin!’”*

-“[If during first 5 test rows subject makes fewer than 21 correct responses, encourage accuracy] – *‘Make sure you don’t go so fast that you miss sixes. Try to be accurate!’* [If subject takes more than about 40 seconds to complete first 5 test rows, encourage rapidity] – *‘Try to go as quickly as you can. It’s all right if you miss a few.’*”

-Stopwatch is started when examiner says “Go”, and stopped when participant vocalizes that he or she has completed each page.

-Record times to the nearest hundredth of a second.

-Participant can go back to cross out missed sixes and review the page. If participant does do this, examiner should say, “Just make sure you tell me when you finish.”

-Discontinue if participant takes 400 or more seconds (6 minutes, 40 seconds) to complete Page 1.

Scoring:

-There are 103 sixes on each page.

-The number of omissions and commissions should be tallied for each page using the clear plastic (overhead) scoring key.

-Record the times and the number of omissions and commissions on both the Digit Vigilance Test administration page and also on the Green Sheet (Neuropsych Scoring – V6) under Item 5 (Digit Vigilance).

-If subtest is discontinued, write the maximum time limit (400 seconds or 6 min 40 sec on Page 1) on the Green Sheet and record the number of errors (omissions/commissions) up to the point of discontinuation.

**Trail Making Test**

Administration: (Directions are located on every set of administration sheets)

-Directions:

Trails A (Sample): *“I want you to draw a line connecting the numbers in order from 1 to 2 to 3 to 4* [trace path from number to number] *and so on, until you reach the end* [point]*. Do it as quickly as you can. Ready? Go.”*

-If participant does not understand or needs more practice, examiner should record the time and errors for each practice trial on the administration sheet.

Trails A (Test): *“Now I want you to do the same thing. This time there are more numbers* [hand Trails A sheet to subject] *so you would connect 1 to 2 to 3 to 4* [very quickly trace path] *and so on all the way to the end at 25* [point]*. Neatness does not count. Remember, work as quickly as you can, and be sure to do the numbers in order. Ready? Go.”*

Trails B (Sample): *“This one is a little different. This one has both numbers and letters and I want you to alternate – number, letter, number, letter. So you would start at 1 and draw a line from 1 to A,* [trace path]*, A to 2, 2 to B, B to 3, 3 to C, and so on, until you reach the end* [point]*. Remember, go number, letter, number, letter as quickly as you can.”*

-If participant does not understand or needs more practice, examiner should record the time and errors for each practice trial on the administration sheet.

Trails B (Test): *“Now I want you to do the same thing. This time there are more numbers and more letters* [hand Trails B sheet to subject]*. Start here at 1 and draw a line from 1 to A, A to 2, 2 to B, B to 3, 3 to C* [trace path]*, and so on until you reach the end at 13* [point to 13]*. Remember to do the numbers and letters in order by alternating number, letter, number, letter. Do this as quickly as you can. Ready? Go.”*

-Stopwatch is started when examiner says “Go.”

-Times are recorded for all trials (even samples) to the nearest hundredth of a second on the Trails administration sheet.

-All tests should be discontinued after 300 seconds (5 minutes).

-If participant makes a mistake, examiner should say “No” and instruct participant to return to the point at which the error was committed. Participant should correct the error and move on.

Scoring:

-Times and the number of errors committed during each trial should be recorded on the Trail Making Test administration sheet and also on the Green Sheet (Neuropsych Scoring – V6) under Item 6 (Trail Making).

-If a trial is discontinued, write the maximum time limit (300 seconds) on the administration sheet and the Green Sheet.

**Stroop Test – AHAB**

"Stroop - Golden Version

This version of the Stroop consists of 3 pages, each with 100 words in 5 columns of 20 items. On page 1(Word card), the words red, green, and blue are presented in black ink. On page 2 (Color card), blocks of X's are printed in red, green, or blue ink. Page 3 (Color-Word card) contains color words printed in non-congruent colors (i.e. the word blue printed in red ink, etc.). The score is the number of correctly identified items per page within 45 seconds. Errors are not counted, but the examiner should point out errors to subject during administration."

Administration: (Directions are located on every set of administration sheets)

-Directions (Words): *“This is a test of how fast you can read the words on this page. After I say ‘Begin’ you are to read down the columns starting with the first one* (point to left column) *until you complete it* (run hand down column) *and then continue without stopping down the remaining columns in order* (run hand down remaining columns)*. If you finish all the columns before I say ‘Stop’ then return to the first column and begin again. Remember, do not stop reading until I say ‘Stop,’ and read out loud as quickly as you can. If you make a mistake, I will say ‘No’ to you. Correct your error and continue without stopping. Are there any questions?* (Instructions may be paraphrased or repeated until subject understands)*. Ready? Go!”*

-Directions (Colors): *“This is a test of fast you can name the colors on this page. You will complete this page just as you did the previous page, starting with the first column. Remember to name the colors out loud as quickly as you can.”*

-Directions (Color/Words): *“This page is like the page you just finished. I want you to name the color of the ink the words are printed in, ignoring the word that is printed in each item. For example,* (point to first item of first column) *this is the first item; what would you say?*

-(If incorrect, examiner should say): *No, that is the word that is spelled there. I want you to say the color of the ink the word is printed in. Try this one* (point to next item)

-(If correct, say) *Good, you will do this page just like the others, starting with the first column* (point) *and then going on to as many columns as you can. Remember, if you make a mistake you must correct it and go on. Are there any questions? Ready, Go!”*

-Stop watch is started when participant says first word.

-Stop watch is stopped at 45 seconds.

-Examiner should follow along with participant on Task 1 (Words), Task 2 (Colors), and Task 3 (Color/Word) administration sheets.

-Examiner should make a mark on each administration sheet at the point in which the task was discontinued (when examiner says ‘Stop’ after 45 seconds of testing). This will tell the examiner the number of items that were completed during each task. This number should be recorded on the administration sheet.

Scoring:

-As noted above, the number of items completed for each task should be recorded on the data administration sheet and also on the Green Sheet (Neuropsych – V6 Scoring) under Item 7 (Stroop) for (Word 45” [Item 7A]), (Color 45” [Item 7D]), and (Color/Word 45” [Item 7G]) respectively.

-If participant is ≥ 45 years of age, the raw scores obtained should be age-corrected. For detailed instructions of this procedure, refer to loose sheet titled Appendix B (Determining T Scores) for Older Adults (45-64) (p. 31)

-In summary, if participant is ≥ 45,

- 8 points should be added to the Words raw score.
- 4 points should be added to the Colors raw score.
- 5 points should be added to the Color/Words raw score.

-T-Scores should be obtained from “Table 1-B T Scores for Stroop Data” on page 31 (Appendix B).

-If participant is younger than 45, the raw scores should be used to determine T Scores.

-If participant is 45 or older, the age-corrected score should be used to determine T Scores.

-If raw score does not exactly match a T-score, score the T-score it’s closer to. Example: Raw score on color trial is 78. Score with the T-score associated with raw score 77 (i.e. 48). If raw score falls in the middle, go with the middle T-score (example raw score on word trial is 98. Available T-scores are 44 (raw=96) or 46 (raw=100). Score as T-score=45.)

- If the T-score falls below 20, then score as 20. If the T-score falls above 99, then score as 99.

- If the participant is color-blind, and the sheets are not administered, record –9999 on the data entry sheet and make a note in the comments section.

-All raw scores, age-corrected scores, and T-Scores should be recorded on Stroop Test – AHAB administration sheet and on the Green Sheet (Neuropsych Scoring – V6) under Item 7 (Stroop) in their proper places.

**General Neuropsych Guidelines:**

**Record all scores on the Neuropsych Scoring Sheet (the Green Sheet) (see attached). Note any behavioral observations on the scoring summary sheet for data entry in the Comments section. For example, subject was tired, color-blind, or uncooperative. Also make sure to note on data entry sheet if the participant is a non-native English speaker. Record participant’s native language and how long he or she has been speaking English on a daily basis.**

1. **DPX** (computer administered)

Follow instructions on hand-out. Using the example “TARGET V. NON-TARGET” card to illustrate the task.

*DPX Task (Dot Pattern Expectancy)*

Access through desktop shortcut

When prompted enter value of 4 for session number followed by participant AHAB-II ID number

I. Instructions:

*During this task you will be asked to press one of these two buttons after each of these patterns (show pattern card to participant).*

*You will press button* ***N (non-target)*** *after all dot patterns you see except this pattern. However, you will only press button* ***T (target)*** *after this dot pattern when it directly follows this dot pattern. If are shown this dot pattern following any other dot pattern, then you will press* ***N*** ***(non-target)****.*

*Clarify by explaining to participant something similar to the following:*

*The only time you should press button* ***T (target)*** *is when you are shown this dot pattern directly after being shown this dot pattern.*

*Remind participant to respond as quickly yet accurately as they can.*

1. **Wechsler Abbreviated Scale of Intelligence** (WASI):

*Next we’ll do a couple of activities that involve defining words and solving problems. Most people don’t answer every item correctly or finish every item, but please give your best effort on all the items. Do you have any questions?*

## WASI SUBTESTS

1. **Block Design**

- Stopwatch is started when tester says go

- Stopwatch is stopped when participant says they are done

- Scramble the blocks between designs

- Make sure to check for rotation errors

- Keep stimulus book approx. 7 inches from and parallel to the edge of the testing table

- See WASI Scoring Manual for additional administration/scoring information

1. **Matrix Reasoning**

- See WASI Scoring Manual

## *- Allow subject to change answer up to the start of the next item*

Note any behavioral observations on the scoring summary sheet for data entry in the Comments section. For example, subject was tired, color-blind or uncooperative.

Also make sure to note if the participant is a non-native English speaker. Record their native language, and how long they have been speaking English on a daily basis.

1. **Food Frequency Interview**

<http://www.nutritionquest.com/login/>

user name: subject ID_2

password: ****

group ID: 268

Upon completion of interview, print copy of report for participant
